# Alternative Receptor Signaling for the Selective and Multifaceted Regulation of Human Brown Adipocytes

**DOI:** 10.3390/ijms27146267

**Published:** 2026-07-14

**Authors:** Yukimasa Takeda

**Affiliations:** Department of Biological and Environmental Chemistry, Faculty of Humanity-Oriented Science and Engineering, Kindai University, 11-6 Kayanomori, Iizuka 820-8555, Fukuoka, Japan; takeday@fuk.kindai.ac.jp

**Keywords:** brown adipose tissue, human brown adipocytes, adipocyte browning, thermogenesis, receptor signaling, G protein-coupled receptors, receptor tyrosine kinases, nuclear receptors, metabolic disorders, therapeutic targets

## Abstract

Brown adipose tissue (BAT) is increasingly recognized as a metabolically active organ in adult humans that contributes to systemic energy homeostasis and represents a potential therapeutic target for obesity-associated metabolic diseases. However, effective strategies to increase BAT mass or thermogenic activity in humans have not yet been established. Although β-adrenergic receptors have traditionally been viewed as the principal drivers of adaptive thermogenesis and adipocyte browning, β-adrenergic stimulation alone may be insufficient to safely enhance BAT thermogenic capacity due to systemic adverse effects. Emerging evidence suggests that alternative receptor-mediated signaling pathways contribute to the regulation of brown adipocyte function, including both UCP1-dependent and UCP1-independent thermogenic mechanisms, and systemic metabolic homeostasis. These pathways include G protein-coupled receptors, receptor tyrosine kinases, and nuclear receptors, which enable brown adipocytes to integrate endocrine, immune, nutritional, and thermal cues. In this review, we discuss recently characterized non-adrenergic receptor signaling pathways and their potential roles in regulating adipocyte browning and thermogenic activity in human adipose tissues. This review highlights the concept of selective modulation of non-adrenergic receptor signaling as a strategy to enhance adipocyte browning and thermogenic capacity while minimizing systemic adverse effects. Understanding these integrated signaling networks may facilitate the development of safer and more selective therapeutic strategies targeting brown adipocyte function in metabolic disease.

## 1. Introduction

### 1.1. Brown Adipose Tissue as a Metabolic Regulator

Obesity and its associated metabolic complications, including insulin resistance, type 2 diabetes, dyslipidemia, and cardiovascular disease, remain major global health challenges [1,2]. Therapeutic strategies have traditionally focused on reducing caloric intake or improving insulin sensitivity; however, increasing energy expenditure represents an attractive complementary approach. In this context, brown adipose tissue (BAT) has emerged as a metabolically active organ capable of dissipating chemical energy as heat and thereby contributing to systemic metabolic regulation [3,4]. Originally regarded as functionally relevant only in rodents and human neonates, BAT was rediscovered in adult humans through imaging studies employing 18F-fluorodeoxyglucose positron emission tomography (FDG-PET) [5,6,7]. These studies demonstrated cold-induced glucose uptake and revealed metabolically active BAT depots in adult individuals, primarily located in the supraclavicular, cervical, and paravertebral regions. These findings indicated that BAT persists into adulthood and is associated with systemic glucose and lipid metabolism [8,9]. Importantly, human BAT differs from rodent BAT in anatomical distribution, developmental origin, receptor expression profiles, and pharmacological responsiveness [10,11]. These species-specific differences underscore the necessity of focusing on human brown adipocyte biology when considering therapeutic translation. Epidemiological analyses have shown inverse correlations between BAT activity and body mass index, blood glucose, and triglycerides, while positively correlating with high-density lipoprotein levels [12]. Accordingly, BAT activity has been associated with a lower prevalence of type 2 diabetes, dyslipidemia, coronary artery disease, cerebrovascular disease, congestive heart failure, and hypertension, suggesting its protective role against obesity-associated metabolic diseases.

The thermogenic capacity of BAT is primarily mediated by uncoupling protein 1 (UCP1), a mitochondrial inner membrane protein that dissipates the proton gradient generated by oxidative phosphorylation [13,14]. Instead of driving ATP synthesis, proton leak through UCP1 releases stored chemical energy as heat, thereby increasing substrate oxidation and energy expenditure. This process not only contributes to cold adaptation but also enhances glucose uptake and fatty acid clearance, positioning BAT as a metabolic sink capable of buffering nutrient excess [15]. Although UCP1 is the canonical mediator of thermogenesis in human brown adipocytes, accumulating evidence indicates the presence of UCP1-independent heat-producing mechanisms [16]. These alternative pathways include Ca^2+^ cycling [17], creatine-driven substrate cycling [18,19,20], futile lipid cycling [21], and peroxisome-dependent futile cycling of monomethyl branched-chain fatty acids (mmBCFA) [22], each capable of dissipating chemical energy as heat without direct reliance on mitochondrial proton leak through UCP1. Receptor-mediated signaling may contribute to the regulation of these pathways as well, suggesting that thermogenic mechanisms in human BAT are more diverse than previously appreciated.

The rediscovery of active BAT in adults has stimulated substantial research interest, particularly because of its potential role in combating obesity and metabolic diseases [23]. Human brown adipocytes exhibit considerable plasticity in response to environmental and hormonal stimuli [15]. Inducible beige (or brite) adipocytes can emerge within white adipose depots under specific physiological conditions [24]. Individuals with higher BAT activity have been shown to display improved glucose tolerance, enhanced insulin sensitivity, and lower body mass index [5,25,26]. Conversely, reduced BAT activity is associated with obesity, aging, and type 2 diabetes [12]. Cold exposure, dietary factors, and pharmacological agents can induce BAT activation [27,28,29], suggesting that brown adipocyte function is tightly regulated by complex signaling networks. These observations have driven growing interest in targeting BAT activation or inducing adipocyte browning as therapeutic strategies for metabolic disorders.

### 1.2. Integration of Cell Surface and Nuclear Receptor Signaling Networks

**Figure 1:**
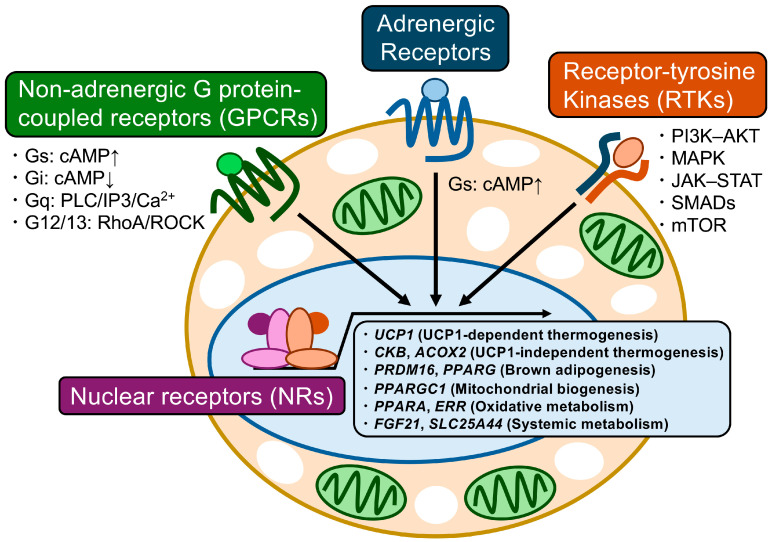
**Overview of alternative receptor signaling regulating adipocyte browning and thermogenic activity in human brown adipocytes.** Human brown adipocytes have been reported to express diverse classes of cell-surface and nuclear receptors beyond β-adrenergic receptors, including G protein-coupled receptors (GPCRs), receptor tyrosine kinases (RTKs), and nuclear receptors (NRs). These alternative receptors engage distinct but interconnected intracellular signaling pathways that regulate UCP1-dependent and UCP1-independent thermogenesis, brown adipogenesis, mitochondrial biogenesis, oxidative metabolism, and systemic metabolism.

Recent transcriptomic analyses of human BAT have identified the expression of multiple receptor classes beyond β-adrenergic receptors. These include G protein-coupled receptors (GPCRs) [30,31], receptor tyrosine kinases (RTKs) [32], and nutrient- and metabolite-sensing nuclear hormone receptors (Figure 1) [33,34]. These receptor systems regulate brown adipocyte function through distinct but interconnected signaling mechanisms that converge on transcriptional networks regulating mitochondrial biogenesis, lipid metabolism, glucose utilization, and thermogenic gene expression. Importantly, dysregulation of these receptor-mediated signaling pathways has been associated with obesity, insulin resistance, and adipose tissue inflammation and metabolic dysfunction. Thus, understanding how these receptor networks contribute to brown adipocyte biology is relevant to the development of targeted interventions.

Membrane receptors can initiate intracellular signaling cascades involving cAMP signaling, calcium signaling, Mitogen-activated protein kinase (MAPK) signaling, phosphatidylinositol 3-kinase (PI3K)–AKT signaling, SMAD signaling, JAK–STAT signaling, and RhoA–Rho-associated coiled-coil containing protein kinase (ROCK) signaling [35,36]. These pathways often converge on nuclear transcriptional regulators such as PPARγ, PGC-1α, PRDM16, and thyroid hormone receptors, which coordinate thermogenic gene programs and mitochondrial biogenesis [37]. These receptor-mediated signaling pathways do not function independently; instead, they frequently interact through cross-talk, feedback regulation, and context-dependent modulation [38]. For example, GPCR activation can enhance mitochondrial gene expression via cAMP–PKA–CREB signaling, whereas growth factor signaling through RTKs may regulate mitochondrial remodeling and oxidative metabolism via PI3K-AKT pathways [39,40]. Nutrient-sensing nuclear receptors adjust cellular metabolism in response to circulating steroids, retinoids, bile acids, and fatty acid metabolites, thereby linking nutrient availability to thermogenic output [41,42]. The modulation of specific receptor pathways may therefore allow more selective enhancement of browning and thermogenesis while minimizing systemic side effects. A comprehensive understanding of receptor subtype expression, downstream signaling specificity, and transcriptional integration within human BAT will be important for future therapeutic development.

### 1.3. Canonical β-Adrenergic Thermogenesis and Species Differences

Cold-induced thermogenesis has traditionally been attributed to sympathetic activation of β-adrenergic receptors (β-ARs) in BAT [43]. In response to cold exposure, the sympathetic nervous system releases norepinephrine, which activates β-adrenergic signaling in brown adipocytes and stimulates thermogenesis through cyclic AMP-dependent pathways [14,15]. In this canonical pathway, norepinephrine binds to Gs-coupled β-ARs on brown adipocytes, leading to activation of adenylyl cyclase, elevation of intracellular cyclic AMP (cAMP), and activation of protein kinase A (PKA). PKA phosphorylates key downstream targets, including hormone-sensitive lipase (HSL) and perilipin, promoting lipolysis and the liberation of fatty acids. These fatty acids serve both as substrates for mitochondrial oxidation and as allosteric activators of uncoupling protein 1 (UCP1), the hallmark thermogenic protein of brown adipocytes. In parallel, PKA activates downstream transcriptional programs involving cAMP response element-binding protein (CREB) and activating transcription factor 2 (ATF2), as well as the transcriptional coactivator PGC-1α that promote thermogenic gene expression and mitochondrial biogenesis.

Despite mechanistic conservation, species differences significantly impact translational relevance. Among the β-AR subtypes (β1, β2, and β3), the relative contribution of each receptor differs between species. While murine BAT exhibits high β3-AR expression and robust thermogenic responsiveness to selective agonists, human BAT appears to rely more heavily on β2-adrenergic signaling than murine BAT, with lower levels of β3-AR expression [11]. Moreover, pharmacological studies demonstrated that administration of a selective β2-agonist stimulated BAT glucose uptake and whole-body energy expenditure in healthy individuals. Consistent with these observations, a recent review discussed the limitations and translational challenges associated with targeting the β3-adrenoceptor for human obesity and type 2 diabetes [44]. These discrepancies highlight the limitations of extrapolating murine findings directly to humans. Providing additional pharmacological evidence in humans, another study demonstrated that salbutamol-mediated β2-adrenergic stimulation increases BAT activity in vivo [45]. Recent genetic evidence also suggests the β2-adrenergic receptor (ADRB2)—rather than β3—as a potential mediator of BAT activation in humans [46]. However, because ADRB2 is broadly expressed in the cardiovascular system, the potential cardiovascular side effects associated with systemic β2-agonism further support the need to develop alternative targeting strategies.

In addition to β-adrenergic receptors, other cAMP-coupled receptor systems have also been implicated in BAT activation. Indeed, adenosine receptors, particularly A2A and A2B subtypes, are expressed in human brown adipocytes and couple primarily to Gs proteins, leading to increased cAMP production [47,48]. Both adenosine receptor subtypes have been shown to potentiate thermogenic activation and enhance UCP1 expression, suggesting a synergistic role with β-adrenergic pathways. In contrast, A1 receptors couple to Gi proteins and may exert inhibitory effects on lipolysis, highlighting subtype-specific regulatory complexity [49]. Similar to β-adrenergic agonists, the clinical translation of targeting adenosine receptors for BAT activation may be limited by potential systemic side effects. Because these receptors are broadly expressed across multiple tissues, their systemic modulation may cause cardiovascular and nervous system effects.

### 1.4. Clinical Limitations of Systemic β-Adrenergic Activation

Despite promising metabolic effects of BAT activation, strategies to safely and effectively increase BAT mass or thermogenic activity in humans remain limited [4,14,50]. Chronic cold exposure can activate BAT and induce the browning of white adipose tissue; however, this approach is challenging to implement and poorly tolerated as a long-term therapeutic intervention [51,52]. Pharmacological activation of BAT using β-adrenergic agonists has therefore been explored as a therapeutic strategy. Although β-adrenergic agonists can acutely enhance BAT activity, clinical application has been constrained by several limitations [52,53]. First, systemic β-adrenergic stimulation can induce tachycardia, hypertension, and arrhythmias, limiting tolerability [54]. Second, β-adrenergic receptor desensitization following chronic stimulation reduces efficacy over time [55]. Third, species differences between rodents and humans complicate translational interpretation, particularly with respect to receptor subtype expression and downstream signaling [10,11]. These challenges highlight the need to identify alternative receptor systems capable of modulating brown adipocyte function without inducing adverse cardiovascular effects. By examining alternative receptor signaling in human BAT, this review aims to provide a conceptual framework for the development of targeted therapies that can reduce dependence on systemic sympathetic activation to modulate human BAT thermogenic function. A deeper mechanistic understanding of these signaling networks will be important for advancing BAT-targeted therapeutic strategies for obesity and related metabolic diseases.

## 2. Non-Adrenergic G Protein-Coupled Receptor Signaling in Human BAT

### 2.1. Expanding the GPCR Landscape Beyond β-Adrenergic Receptors

G protein-coupled receptors (GPCRs) constitute the largest family of membrane receptors in mammalian cells and regulate diverse physiological processes, including metabolism, inflammation, and endocrine signaling [31,56]. While β-adrenergic receptors represent the canonical GPCR mediators of thermogenesis, transcriptomic analyses of human BAT and primary brown adipocytes have identified numerous additional GPCR subtypes [30,57,58]. These receptors respond to ligands such as prostaglandins, purines, peptide hormones, bile acids, chemokines, fatty acids, and phospholipids, suggesting that brown adipocytes respond to a broad range of extracellular cues beyond sympathetic stimulation. These GPCRs signal through distinct heterotrimeric G proteins, including Gs, Gi, Gq, and G12/13, which can activate intracellular pathways such as cAMP–PKA, phospholipase C (PLC)–inositol trisphosphate (IP_3_)–Ca^2+^ signaling, MAPK cascades, and RhoA–ROCK [31]. The diversity of GPCR coupling likely enables context-dependent regulation of mitochondrial function, energy metabolism, and thermogenic transcriptional programs. Importantly, targeting GPCRs enriched in human brown adipocytes may provide opportunities to modulate adipocyte browning and thermogenic activity with greater tissue selectivity than systemic β-adrenergic stimulation, potentially minimizing cardiovascular side effects (Table 1).

### 2.2. Prostaglandin Receptors

Prostaglandins derived from arachidonic acid regulate adipose tissue remodeling and thermogenic function [90]. Among prostaglandin receptors, the Gs-coupled EP2 and EP4 receptors promote cAMP production and have been implicated in adipocyte browning, mitochondrial biogenesis, and UCP1 expression [61,91,92]. In contrast, the Gi-coupled EP3 receptor generally suppresses cAMP signaling and thermogenic pathways, although recent evidence suggests additional roles in BAT differentiation through post-transcriptional mechanisms [59,60]. These findings highlight the importance of receptor subtype-specific effects within the PGE2 signaling network.

Other prostanoid pathways also influence adipocyte plasticity. Prostacyclin (PGI2) signaling through the IP receptor promotes browning of human white adipocytes via cAMP-dependent mechanisms [63,64], whereas thromboxane A2 (TXA2) has been reported to antagonize these pro-thermogenic effects [93]. In contrast, PGF2α signaling through the FP receptor suppresses browning and UCP1 expression via calcium- and ERK-dependent pathways [65]. Together, these findings indicate that prostanoid receptor signaling exerts both positive and negative effects on thermogenic adipocyte function and may represent a targetable non-adrenergic regulatory pathway [66].

### 2.3. Purinergic Receptors

Extracellular nucleotides released during cellular stress or sympathetic co-transmission activate purinergic ligand-gated ion channels (P2X receptors) and GPCRs (P2Y receptors). In brown adipocytes, purinergic signaling has been implicated in regulating mitochondrial activity and metabolic adaptation to stress [49]. Although P2RX5 is a ligand-gated ion channel rather than a GPCR, it is discussed here because purinergic signaling in BAT involves coordinated actions of both P2X and P2Y receptor families. The P2RX5 receptor shows enriched expression in mature brown adipocytes isolated from BAT and has been identified as a novel modulator of glucose homeostasis and thermogenic gene expression in brown adipocytes [94,95].

As purinergic GPCRs, the Gq-coupled P2RY2, P2RY4, and P2RY6 receptors—activated by specific nucleotides such as ATP, UTP, and UDP—may function as metabolic sensors [67]. In brown adipocytes, activation of these P2RY receptor subtypes can differentially regulate intracellular calcium mobilization, contributing to metabolic responses [96]. P2Y11 (P2RY11) is a human-specific purinergic GPCR activated by extracellular ATP that uniquely couples to both Gs and Gq proteins, leading to activation of cAMP and Ca^2+^ signaling pathways [68,69,97]. Transcriptomic analyses indicated that the pro-thermogenic hormone T3 upregulates the transcription of the P2RY11 receptor, potentially interacting with cAMP–PKA-dependent thermogenic signaling. P2RY11 may modulate both metabolic and inflammatory signaling in human tissues and has been proposed as a potential regulator of human adipocyte browning. However, current knowledge regarding P2 receptors in human BAT remains scarce, necessitating further comprehensive studies to elucidate the exact underlying mechanisms.

### 2.4. Glucagon and Incretin Receptors

The glucagon receptor (GCGR) signaling can stimulate energy expenditure and brown adipocyte thermogenesis by activating cAMP-dependent pathways in adipose tissue [98]. Collectively, these findings support the potential of targeted GCGR agonists, particularly in the context of novel multi-receptor (GLP1R/GIPR/GCGR) co-agonists (e.g., Retatrutide), as a strategy for metabolic intervention involving BAT-associated pathways. Supporting this pathway, a study demonstrated that the loss of endogenous glucagon signaling impairs the browning of white adipose tissue [70]. The researchers showed that disrupting GCGR signaling downregulated UCP1 expression and blunted cold-induced browning of WAT. These findings suggest that endogenous GCGR signaling contributes to adipocyte plasticity and thermogenic remodeling. Although modern multi-receptor therapies yield systemic metabolic benefits, their direct actions on adipocyte thermogenesis are receptor-specific. A study that demonstrated the direct activation of BAT and localized energy expenditure achieved by Glucagon-like peptide 1 receptor (GLP1R)/GCGR dual agonism appeared to be largely attributable to GCGR signaling [71]. A recent report suggested relatively limited GLP1R expression in primary human adipocytes compared with Glucose-dependent insulinotropic polypeptide receptor (GIPR) [72], although GLP1R agonist liraglutide promotes mitochondrial respiration and biogenesis through GLP1R expressed in human SGBS adipocytes [99]. This functional distribution suggests that the GCGR may contribute more directly to adipocyte thermogenic regulation than GLP1R.

Consistent with the limited direct role of GLP1R in adipose tissue, a recent study evaluating the dual agonist tirzepatide for GLP1R/GIPR revealed that its direct regulation of adipocyte nutrient metabolism is primarily mediated by the GIP receptor [72]. Because mature adipocytes show limited functional GLP1R signaling, the metabolic remodeling associated with incretin therapies relies in part on GIPR signaling. Together with GCGR, these findings suggest that GIPR signaling may contribute to adipocyte metabolic regulation and remodeling. Another study demonstrated that acute administration of exogenous acyl-GIP enhances lipid handling and fatty acid oxidation specifically by engaging brown fat [73]. These findings indicate that GIPR signaling may contribute to thermogenic lipid metabolism independent of GLP-1R. Collectively, GCGR and GIPR signaling pathways may represent potential non-adrenergic mechanisms that influence cAMP-associated thermogenic and metabolic regulation.

### 2.5. Bile Acid Receptors

Bile acids function not only in lipid digestion but also as endocrine signaling molecules [100]. Brown adipocytes express the bile acid receptor TGR5 that couples to Gs proteins, supporting cAMP production and promoting thermogenic gene expression [101]. Acute administration of chenodeoxycholic acid (CDCA) has been shown to enhance BAT activity and whole-body energy expenditure in humans. Mechanistically, the binding of bile acids to TGR5 triggers the intracellular cAMP-PKA signaling pathway, a cascade that promotes the expression of UCP1 and type 2 deiodinase (DIO2), thereby potentially amplifying local thyroid hormone signaling. Supporting a thermogenic role for bile acid signaling in vivo, a complementary study revealed that bile acids induce UCP1-dependent thermogenesis and elevate energy expenditure in mice even under thermoneutral conditions [102]. Together, these findings suggest that the TGR5/cAMP/PKA signaling axis contributes to the regulation of adipocyte browning and systemic energy metabolism. In addition to its direct effects on thermogenic signaling, TGR5 also influences systemic metabolism through adipose–liver crosstalk [103]. This study indicates that the loss of adipocyte TGR5 impairs thermogenic activity and significantly reduces the secretion of adiponectin, an insulin-sensitizing hormone. The resulting decline in adiponectin levels may exacerbate hepatic steatosis. In a recent study, a secondary analysis of the ACTIBATE study indicates that cold-induced changes in circulating bile acid levels were significantly associated with BAT activity and volume in humans [74]. These findings are consistent with a potential contribution of bile acid signaling to adaptive thermogenic response to cold stress in a human cohort. In obesity, altered bile acid composition and impaired receptor responsiveness may blunt these thermogenic effects.

### 2.6. Chemokine Receptors

Chemokine receptors, many of which couple to Gi proteins, are frequently upregulated in inflamed adipose tissue [104]. Chemokine receptors are predominantly expressed in immune cells within adipose tissue; however, several receptors, including CXCR4 and CCR5, are also expressed in human adipocytes and have been implicated in the regulation of thermogenic signaling [75,77]. Activation of these receptors can initiate MAP kinase and NF-κB signaling, promoting inflammatory gene expression while potentially impairing mitochondrial function and thermogenic activity. Chronic activation of proinflammatory chemokine receptors in obesity may contribute to impaired browning capacity by suppressing cAMP-dependent transcriptional programs. Augmented CCL5/CCR5 signaling has been found to inhibit adaptive thermogenesis in BAT [75]. This study suggests that in the context of obesity, the overactivation of the CCL5-CCR5 pathway is associated with impaired UCP1 expression and worsened insulin resistance. The CXCL12-CXCR4 signaling axis has been proposed as a non-adrenergic regulator of brown fat thermogenic capacity and systemic metabolic health. Research using adipocyte-specific models demonstrated that disruption of CXCL12–CXCR4 signaling impairs BAT thermogenic function, reduces UCP1 expression, and exacerbates diet-induced metabolic dysfunction under high-fat diet conditions [76,77]. Together, these findings suggest that CXCR4 may function as a protective regulator of thermogenic function that preserves mitochondrial function and glucose homeostasis during metabolic stress. Thus, selective modulation of chemokine receptors expressed in brown adipocytes may represent a strategy to improve thermogenic function in dysfunctional BAT, as different receptor subtypes exert either inhibitory or protective effects.

### 2.7. Angiotensin Receptors

The renin–angiotensin system (RAS) has also been implicated in the regulation of metabolic homeostasis beyond its classical cardiovascular roles. In adipose tissues, locally produced angiotensin II acts through the angiotensin II type 1 receptor (AGTR1, AT1R) and the angiotensin II type 2 receptor (AGTR2, AT2R) [105]. A recent study suggests that Ang II signaling, through its type 1 receptor (AT1R), increases glycolytic flux, which may provide metabolic substrates for enhanced mitochondrial respiration and UCP1-mediated heat production [78]. These findings suggest that Ang II signaling links systemic cardiovascular regulation with brown adipocyte metabolic reprogramming. Beyond its role in cardiovascular homeostasis, the ACE2-Angiotensin-(1–7) signaling axis has been proposed as a non-adrenergic regulator of thermogenesis and energy expenditure. Several studies have suggested that ACE2 serves as an upstream regulator of metabolic rate and thermoregulation [106], in part by generating Angiotensin-(1–7). This peptide, in turn, has been reported to promote the browning of white adipose tissue and thermogenic gene expression through the Mas receptor in beige and brown adipocytes [79]. Together, these findings suggest that the ACE2–Angiotensin-(1–7)–Mas axis may contribute to thermogenic regulation and metabolic homeostasis beyond classical β-adrenergic signaling.

### 2.8. Free Fatty Acid Receptors (FFARs)

Free fatty acids (FFAs) are both substrates and signaling molecules in brown adipocytes. Free fatty acid receptors may act as lipid sensors, modulating brown adipocyte activity in response to circulating nutrient levels [107]. In addition to activating UCP1 within mitochondria, FFAs bind to membrane receptors that modulate intracellular signaling pathways. The free fatty acid receptor 4 (GPR120, also known as FFAR4) has emerged as a potential non-adrenergic modulator of adipocyte biology [80]. Activated primarily by long-chain omega-3 polyunsaturated fatty acids, GPR120 signaling has been proposed to modulate thermogenic programs through intracellular calcium mobilization and the regulation of UCP1 expression, potentially in a manner partially independent of classical β-adrenergic signaling. The pharmacological regulation of GPR120 via the agonist TUG-891 triggers intracellular calcium release, associated with mitochondrial depolarization and fission [107,108]. This shift in mitochondrial dynamics has been associated with increased uncoupled respiration, potentially through mechanisms distinct from classical adrenergic signaling, suggesting a promising therapeutic strategy to enhance mitochondrial thermogenesis. Recent evidence expands this regulatory axis by demonstrating that hepatic ACOX1 activity influences the levels of specific circulating signaling lipids, which act as endogenous ligands to promote adipose remodeling and thermogenic function via GPR120 [81].

The orphan receptor GPR3 has been identified as a non-adrenergic regulator of energy expenditure due to its unique constitutive activity. Early studies demonstrated that GPR3-deficient mice develop late-onset obesity characterized by significantly impaired thermogenic function in BAT [82]. Building on this, recent research reveals that GPR3 expression is dynamically driven by intracellular lipolysis, creating a feed-forward loop that sustains UCP1-mediated thermogenesis independently of continuous β-adrenergic input [83]. Further identifying lipid-sensing mechanisms, the medium-chain fatty acid receptor GPR84 has been implicated in the regulation of metabolic function and brown adipocyte activity [86]. Although initially demonstrated to regulate mitochondrial metabolism in skeletal muscle, the medium-chain fatty acid receptor GPR84 may also enhance mitochondrial function and thermogenesis in brown adipocytes [85].

### 2.9. Sphingosine 1-Phosphate Receptors (S1PRs)

Bioactive sphingolipids are emerging as modulators of adipose health, particularly in the context of aging and overnutrition. Altered sphingolipid metabolism has been associated with BAT dysfunction during aging [88]. In parallel, the S1P2 receptor signaling axis has been implicated in obesity-associated metabolic deterioration; its blockade has been shown to attenuate adipocyte hypertrophy and glucose intolerance induced by a high-fat diet [87]. Together, these findings suggest that sphingolipid signaling may contribute to adipose tissue dysfunction and metabolic impairment, highlighting this pathway as a potential non-adrenergic target for further investigation. In contrast to the detrimental role of S1P2, the S1P/S1PR3 signaling axis has been proposed as a protective mechanism against obesity-induced metabolic dysfunction [89]. This study suggests that S1PR3 activation was associated with increased thermogenic gene expression and improved insulin sensitivity, indicating differential roles within sphingosine-1-phosphate signaling. By selectively engaging the S1PR3 pathway, S1PR3 signaling may contribute to the maintenance of adipose metabolic function under conditions of metabolic stress.

### 2.10. Therapeutic Implications of Targeting Non-Adrenergic GPCRs

Non-adrenergic GPCRs broaden the regulatory network of human brown adipocytes by integrating hormonal, immune, and metabolic signals through diverse G protein-mediated pathways, potentially interacting with β-adrenergic signaling and other receptor systems (Table 1) [30]. Gi-coupled receptors can attenuate β-adrenergic cAMP generation, whereas Gq-mediated calcium signaling can interact with cAMP-dependent pathways to modulate mitochondrial function and thermogenic gene expression [69,97]. Dysregulation of GPCR signaling has been associated with impaired BAT function in obesity, while selective targeting of specific GPCR subtypes represents a promising strategy to enhance browning and metabolic health, potentially with reduced adverse effects.

Obesity has been associated with altered expression of multiple GPCR subtypes in adipose tissues, including BAT [30,109]. Chronic nutrient excess, low-grade inflammation, and hyperinsulinemia can shift GPCR signaling toward inhibitory or inflammatory pathways, thereby disrupting metabolic regulation. Reduced cAMP responsiveness, enhanced chemokine receptor activation, and impaired mitochondrial signaling have each been associated with diminished thermogenic capacity [75,110]. However, significant challenges remain, including receptor redundancy, broad tissue distribution beyond BAT, and species-specific differences in receptor expression patterns. Rigorous characterization of human BAT-specific GPCR signatures will therefore be essential for successful therapeutic translation.

## 3. Receptor Kinase Signaling in Human BAT

### 3.1. RTKs as Regulators of Adipocyte Differentiation and Metabolic Plasticity

Receptor tyrosine kinases (RTKs) constitute a major class of membrane receptors that regulate cell growth, differentiation, metabolism, and survival [111]. Unlike GPCRs, which primarily modulate second messenger systems, RTKs initiate signaling through ligand-induced receptor dimerization and autophosphorylation of intracellular tyrosine residues. These phosphorylated residues serve as docking sites for adaptor proteins and enzymes, triggering cascades such as phosphoinositide 3-kinase (PI3K)-Akt, Ras-MAPK, phospholipase Cγ (PLCγ), and Janus kinase (JAK)-STAT pathways. In brown adipocytes, RTK signaling contributes to early adipogenic commitment while also modulating mitochondrial function, oxidative metabolism, and thermogenic responsiveness [32,112]. Altered RTK signaling has been implicated in BAT dysfunction associated with obesity and insulin resistance. Several RTK-associated pathways converge on transcriptional regulators central to brown adipocyte identity. Downstream signaling pathways, such as PI3K-Akt and MAPK, influence the activity and stability of transcriptional regulators such as PPARγ, PGC-1α, PRDM16, and FOXO [113]. Akt-mediated phosphorylation of FOXO proteins excludes them from the nucleus, modulating oxidative gene expression [114]. MAPK signaling can phosphorylate PPARγ, altering its transcriptional activity [115]. Furthermore, RTK signaling can activate mTORC1, which regulates protein synthesis, ribosomal biogenesis, and mitochondrial metabolic programs. Although much of the mechanistic evidence derives from rodent or in vitro models, emerging transcriptomic and functional studies suggest similar RTK networks may also function in human BAT. Through these mechanisms, RTKs integrate extracellular growth and nutrient signals with transcriptional programs that regulate brown adipocyte differentiation, metabolic activity, and thermogenic capacity (Table 2).

### 3.2. Insulin Receptor Signaling: Metabolic Support for Thermogenesis

The insulin receptor (IR) is a prototypical RTK that regulates glucose uptake and anabolic metabolism [131]. Upon insulin binding, autophosphorylation of the receptor recruits insulin receptor substrates (IRS proteins), which activate PI3K and subsequently Akt. Akt promotes translocation of glucose transporter 4 (GLUT4) to the plasma membrane, enhancing glucose uptake. In brown adipocytes, glucose serves as an important substrate for both glycolysis and de novo lipogenesis, providing intermediates that support mitochondrial respiration and may facilitate thermogenic activity [132]. Akt signaling influences mitochondrial function indirectly through modulation of transcription factors such as FOXO and through activation of mTOR complexes [35]. Balanced mTOR signaling is important for mitochondrial biogenesis and protein synthesis; however, chronic hyperactivation may promote lipogenesis at the expense of oxidative metabolism [133]. In obesity, insulin resistance impairs PI3K-Akt signaling in adipose tissue, including BAT [134,135]. Reduced Akt activation diminishes glucose uptake and may compromise mitochondrial substrate flexibility. Furthermore, impaired insulin signaling can alter FOXO localization, affecting transcriptional programs that regulate the balance between oxidative metabolism and lipid storage [136]. Understanding this canonical insulin-mediated nuclear exclusion of FOXO provides a conceptual framework for evaluating how alternative receptor pathways might rewire lipid and glucose metabolism in human brown adipocytes. In addition, chronic hyperinsulinemia may induce receptor desensitization and promote inflammatory signaling pathways, which may reduce thermogenic capacity in BAT [137]. A previous study demonstrated that the fat-specific deletion of both insulin and IGF-1 receptors leads to a near-complete failure of BAT development and severe cold intolerance [116]. This study underscores that while adrenergic signaling triggers thermogenesis, insulin and IGF-1 receptor-mediated pathways provide essential metabolic support for brown adipocyte differentiation and development. These pathways also contribute to the induction of thermogenic programs including UCP1 expression. Although much of this evidence derives from studies in rodents and cultured adipocytes, insulin signaling is also active in human BAT and is thought to support substrate utilization and metabolic flexibility in vivo.

### 3.3. Fibroblast Growth Factor Receptors (FGFRs): Endocrine Control of Browning

Fibroblast growth factor 21 (FGF21) is an endocrine hormone that exerts pleiotropic metabolic effects associated with increased energy expenditure and promotion of adipose tissue browning [138]. FGF21 signals primarily through FGFR1c in complex with the obligate co-receptor β-Klotho (KLB) [139]. Activation of FGFRs triggers intracellular phosphorylation cascades involving the MAPK/ERK and PI3K–Akt pathways, which regulate metabolic gene expression. In brown adipocytes, FGF21 enhances expression of thermogenic genes, including UCP1 and PGC-1α, and promotes mitochondrial biogenesis and oxidative metabolism in mice [140]. FGF21 signaling may also increase fatty acid oxidation and improve systemic insulin sensitivity, supporting its role as an important endocrine regulator of BAT activity. In human BAT, FGFR1 and β-Klotho are expressed, although the functional contribution of FGF21 signaling remains less well defined compared with rodent models.

BAT itself can produce FGF21 during thermogenic stimulation, suggesting potential autocrine or paracrine amplification mechanisms within BAT [141]. In obesity and metabolic disease, circulating FGF21 concentrations are often elevated, yet target tissue responsiveness appears reduced, a phenomenon often referred to as “FGF21 resistance.” Recent findings suggest that FGF21 has been proposed as an important non-adrenergic mediator that contributes to the browning of white adipose tissue [32,142]. In this context, FGF21 enhances UCP1 expression and thermogenic capacity during cold exposure through the FGFR1-β-Klotho complex. These findings suggest that FGF21 acts synergistically with classical sympathetic activation. Understanding FGFR expression levels and downstream signaling integrity in human BAT is therefore important for the development of therapeutic strategies targeting the FGF21–FGFR–β-Klotho axis to enhance thermogenic activity.

### 3.4. Epidermal Growth Factor Receptor (EGFR) and Related Pathways

Epidermal growth factor receptor (EGFR) and other members of the ErbB receptor tyrosine kinase family regulate diverse cellular processes including proliferation, differentiation, and survival [143]. In pathological conditions such as obesity, dysregulated EGFR signaling within the adipose niche has been associated with adipose tissue inflammation, remodeling, and fibrosis, processes that may indirectly impair BAT thermogenic capacity [117]. These findings suggest that EGFR signaling represents a potentially detrimental non-adrenergic pathway within the adipose niche, where its activation in macrophages promotes chronic inflammation and contributes to the development of obesity and insulin resistance. These findings highlight that RTK signaling in non-adipocyte immune cells within the BAT niche can indirectly influence overall adipose tissue thermogenesis and metabolic health.

Neuregulin 4 (NRG4) has emerged as an important batokine that links thermogenic adipose tissue to systemic metabolic health [144]. NRG4 is expressed in human adipose tissue and has been proposed as a marker of brown/beige adipocytes; its expression is positively correlated with UCP1 levels [145]. ErbB4, a member of the EGFR (ErbB) superfamily of receptor tyrosine kinases, is the primary receptor for NRG4. The genetic deletion of ErbB4 in mice is associated with increased susceptibility to metabolic dysfunction, characterized by increased adiposity and impaired glucose homeostasis [118]. These findings support a role for the NRG4–ErbB4 axis in systemic metabolic regulation. They also suggest that this pathway contributes to the regulation of brown adipocyte function, including in human BAT. A recent study further expands this role, demonstrating that NRG4 has also been shown to mediate the metabolic benefits of mild cold exposure by promoting beige fat thermogenesis [119]. Together, these studies suggest that the NRG4–ErbB4 signaling axis functions as an important non-adrenergic regulator that may coordinate both local thermogenic activity and inter-organ crosstalk.

### 3.5. Transforming Growth Factor-β (TGF-β) Superfamily Signaling

Members of the TGF-β superfamily, including activins and bone morphogenetic proteins (BMPs), signal through serine/threonine kinase receptors and Smad transcription factors [146]. Although structurally distinct from classical cytokine receptors, these pathways play key roles in adipose tissue remodeling. TGF-β signaling can inhibit adipocyte differentiation and promote fibrosis [147]. Elevated TGF-β levels in obesity contribute to extracellular matrix deposition and impaired tissue plasticity, potentially limiting browning capacity. The TGF-β signaling pathway functions as a negative regulator of thermogenic adipocyte development and tissue health. A previous study demonstrated that pharmacological inhibition of the TGF-β receptor via the small molecule RepSox promotes brown adipogenesis and white fat browning [120]. Recent evidence suggests a synergistic interaction: TGF-β antagonism not only enhances beige adipogenesis but also cooperates with PPARγ activation to alleviate adipose tissue fibrosis [121]. Together, these studies suggest that inhibition of TGF-β signaling may represent a dual-action strategy to enhance thermogenic capacity while remodeling the fibrotic, dysfunctional environment of obese adipose tissue.

Conversely, BMPs have been shown to promote brown adipocyte differentiation and mitochondrial gene expression. Activation of BMP receptors stimulates Smad1/5/8 signaling, enhancing expression of thermogenic transcriptional regulators [146,148]. In progenitor cells, BMP signaling biases lineage commitment toward brown adipocytes. BMPs represent a distinct class of non-adrenergic regulators that modulate both adipocyte identity and functional output. A recent study showed that BMP4 and BMP9 exhibit depot-specific signaling dynamics that distinguish brown from white adipocyte responses [122]. BMP4 and BMP9 were found to control a specialized transcriptional program in brown adipocytes that is not fully replicated in white adipocytes. These findings suggest that a specialized epigenetic landscape contributes to the selective amplification of BMP signaling toward a thermogenic fate. Subsequent findings indicate that BMP4 and BMP7 act as important non-adrenergic regulators of thermogenesis in human adipose tissues [123]. Notably, this study utilized primary human adipocytes to show that treatment with these proteins increases UCP1 expression and enhances mitochondrial respiratory capacity. Collectively, these studies suggest the therapeutic potential of fine-tuning BMP signaling dynamics to enhance thermogenic activity in human adipose tissue. TGF-β signaling through TGFBR1/2 suppresses browning and thermogenic gene expression, whereas BMP signaling via BMPR1A/BMPR2 promotes brown adipocyte differentiation and mitochondrial biogenesis. Together, these pathways illustrate the balance between inhibitory and activating growth factor signaling in thermogenic adipocytes. In human adipose tissue, components of the TGF-β superfamily are expressed, and balanced TGF-β superfamily signaling likely contributes to maintaining BAT functionality.

### 3.6. Platelet-Derived Growth Factor Receptors (PDGFRs) and Adipose Lineage Commitment

PDGFR signaling influences mesenchymal stem cell fate decisions, modulating differentiation toward adipogenic versus fibrotic lineages [149]. While much focus has been placed on pro-thermogenic pathways, the PDGFR signaling axis represents a non-adrenergic regulatory pathway that influences adipose tissue thermogenesis. A previous study provided insights into how the PDGFRα signaling pathway regulates the pathological remodeling of adipose tissue [124]. Their research demonstrated that sustained activation of PDGFRα in adipose progenitor cells (APCs) induces a shift in cellular plasticity. As a result, APCs transition from an adipogenic phenotype toward a pro-fibrotic, myofibroblast-like state. Another study demonstrated vascular-adipose crosstalk, showing that angiogenic endothelial cells contribute to the regulation of white adipose tissue browning by secreting PDGF-CC [125]. This study demonstrates that endothelial-derived PDGF-CC signals through PDGFRα on adipose progenitor cells to increase UCP1 and promote beige adipocyte differentiation. While chronic PDGFRα activation is classically associated with fibrosis, the specific, angiogenesis-coupled delivery of PDGF-CC may function as a non-adrenergic regulator for thermogenesis. These findings suggest that the biological outcome of PDGFRA signaling is highly context-dependent. In thermogenic fat, this fibrotic transformation appears to be detrimental; it creates a microenvironment that impairs the recruitment and functional expansion of UCP1-positive beige and brown adipocytes.

Recent lineage-tracing studies have redefined the origins of thermogenic fat, highlighting the perivascular niche as an important cellular reservoir [126]. This study showed that Trpv1-expressing vascular smooth muscle cells—a subpopulation within the PDGFRβ-expressing perivascular lineage—serve as a distinct source of cold-induced brown adipocytes. Expanding the understanding of developmental constraints, another recent study suggested that the Notch-PDGFRβ axis functions as an inhibitory mechanism that suppresses the differentiation of brown adipocyte progenitors during the early postnatal phase [127]. This research suggests that the interplay between Notch and PDGFRβ signaling acts as a regulatory mechanism, maintaining the progenitor pool in an undifferentiated, quiescent state and preventing premature thermogenic activation.

### 3.7. Vascular Endothelial Growth Factor Receptors (VEGFRs): Coupling Angiogenesis and Thermogenesis

BAT is highly vascularized, reflecting its substantial oxygen and nutrient demands required for sustained thermogenic activity [150]. Vascular endothelial growth factor (VEGF) signaling through VEGF receptors (VEGFRs) promotes angiogenesis and enhances blood flow within BAT depots. Increased vascularization improves delivery of oxygen and metabolic substrates, thereby supporting mitochondrial respiration and thermogenesis. VEGF signaling may support BAT thermogenesis indirectly through vascular remodeling and modulation of the local adipose microenvironment. An early mouse study demonstrated that BAT-derived VEGF-A plays an important role in promoting local angiogenesis to meet the increased oxygen and nutrient demands during cold-induced thermogenesis [128]. In addition, a recent study suggests a neuro-immune mechanism for VEGF-B in mice [129]. This study provides evidence suggesting that VEGF-B is associated with increased thermogenesis by modulating adipose tissue macrophage function, thereby reducing norepinephrine clearance. VEGF signaling components are expressed in human BAT, suggesting potential relevance to human thermogenic regulation.

### 3.8. AXL Signaling: An Emerging Negative Regulator of Thermogenesis

AXL is a member of the TAM (Tyro3, AXL, Mer) family of receptor tyrosine kinases, primarily activated by its ligand GAS6 [151]. A recent study identified the AXL receptor tyrosine kinase as a negative regulator of thermogenesis in mice [130]. This study demonstrated that either genetic ablation or pharmacological inhibition of AXL enhances BAT thermogenic activity and systemic energy expenditure in mice. Consequently, the potential repurposing of AXL inhibitors—which are already undergoing clinical evaluation in oncology—represents a potential therapeutic approach to relieve inhibitory constraints on brown adipocyte activation, although further validation in human BAT and clinical settings is required.

### 3.9. Therapeutic Potential of Targeting RTKs in BAT

Dysregulated growth factor signaling in obesity may favor adipose tissue expansion over thermogenic remodeling. Targeting RTK pathways offers several potential translational opportunities. NRG4–ERBB4 signaling influences systemic metabolism and has been implicated in the regulation of BAT innervation and metabolic activity, suggesting that modulation of this pathway may enhance BAT thermogenic capacity and systemic metabolism [119]. Activation of TGF-β/SMAD pathways promotes adipose tissue fibrosis and suppresses beige adipocyte differentiation, whereas pharmacological inhibition of TGF-β receptor signaling has been shown to enhance beige adipogenesis and improve metabolic parameters in experimental models [120]. In contrast, activation of the BMP–BMPR axis represents a potential strategy to enhance the recruitment of thermogenic adipocytes and improve systemic energy metabolism [123]. PDGFR-positive progenitor populations contribute to beige adipocyte formation. These findings suggest that PDGFR signaling may represent a therapeutic target linking angiogenesis, progenitor cell activation, and thermogenic adipocyte recruitment. Moreover, targeting AXL with specific antagonists represents a potential therapeutic approach to enhance BAT activity [130]. Collectively, these RTK pathways highlight multiple avenues to therapeutically modulate thermogenic adipose tissue. However, much of the current evidence derives from rodent models, and further studies are required to validate these strategies in human BAT. Strategies that activate pro-thermogenic receptor signaling pathways (FGFR1, ERBB4, BMPR) or inhibit anti-thermogenic signaling pathways (TGFBR, AXL) may enhance BAT activity and energy expenditure, providing a conceptual framework for future anti-obesity strategies.

## 4. Nuclear Receptor Integration and Transcriptional Networks in Human BAT

### 4.1. Nuclear Receptors as Key Integrators of Thermogenic Identity

While membrane receptors initiate rapid signaling cascades in brown adipocytes, nuclear receptors (NRs) play central roles in coordinating transcriptional reprogramming associated with the maintenance of brown adipocyte phenotype and thermogenic capacity [33,34,152]. These ligand-activated NRs function as metabolic sensors, integrating hormonal, nutrient, and intracellular cues into coordinated gene expression programs that regulate oxidative metabolism, mitochondrial biogenesis, and adaptive thermogenesis [153]. Acting as ligand-activated transcription factors, nuclear receptors bind specific DNA response elements and recruit coactivators or corepressors to regulate transcription of metabolic genes. In human BAT, nuclear receptors likely integrate endocrine signals—including thyroid hormones, glucocorticoids, retinoids, and bile acids—as well as nutrient-derived fatty acids and related metabolites. These inputs are further modulated by intracellular pathways downstream of GPCRs and RTKs, such as PI3K–Akt and MAPK [154]. In turn, NR activity is regulated through post-translational modifications and cofactor recruitment, enabling coordinated regulation of mitochondrial biogenesis, lipid oxidation, glucose utilization, and UCP1 expression. Importantly, nuclear receptor signaling contributes to the activation of thermogenic gene programs and the maintenance of brown adipocyte lineage identity and may limit reversion toward white adipocyte-like phenotypes (Table 3).

### 4.2. Peroxisome Proliferator-Activated Receptor γ (PPARγ): Central Regulator of Adipocyte Identity

PPARγ is essential for adipocyte differentiation in both white and brown adipose lineages [34]. In brown adipocytes, PPARγ cooperates with transcriptional cofactors such as PRDM16, EBF2, and PGC-1α to promote thermogenic gene expression, including UCP1 and mitochondrial oxidative programs [153]. Pharmacological activation of PPARγ can induce browning in human adipocyte models [176,177], although its therapeutic application is limited by systemic adverse effects. In addition, interactions between PPARγ and RXRγ have been implicated in the regulation of adipocyte browning and thermogenic transcriptional programs [166]. Thus, PPARγ contributes to the regulation of brown adipocyte identity and thermogenic function through interactions with brown fat-selective transcriptional cofactors.

### 4.3. PPARα and PPARδ: Drivers of Oxidative Metabolism

PPARα and PPARδ support thermogenic programs by promoting fatty acid oxidation and mitochondrial oxidative metabolism in brown adipocytes [155]. PPARα contributes to thermogenic gene regulation through the control of PGC-1α and other metabolic regulators, thereby supporting thermogenic capacity in BAT [156,157]. PPARδ also promotes oxidative remodeling and can be activated by lipid-derived signals generated through nutrient-sensing pathways such as the VLDL–VLDLR axis, linking extracellular lipid availability to transcriptional programs associated with thermogenic function [158]. Together, PPARα and PPARδ function as metabolic regulators that support oxidative and thermogenic programs in brown adipocytes.

### 4.4. Liver X Receptors (LXRs)

In addition to classical regulators such as PPARs and retinoid receptors, other lipid- and metabolite-sensing nuclear receptors may contribute to the transcriptional network governing brown adipocyte metabolism [178]. Liver X receptors (LXRα/β), which are activated by oxysterols, are key regulators of cholesterol and lipid homeostasis and can influence lipogenic gene expression through LXR–RXR transcriptional complexes. Although LXR signaling has been associated with increased lipid storage programs, its interaction with PPAR-dependent transcriptional pathways suggests a context-dependent role in shaping substrate utilization and thermogenic metabolism in brown adipocytes [179]. Pharmacological activation of LXR suppresses UCP1 expression and adipocyte browning, indicating that LXR signaling negatively regulates thermogenic gene programs [159,160]. Consistently, previous work provided evidence that the loss of both LXR isoforms resulted in increased energy expenditure and BAT activity [159]. Subsequent research suggested that LXRβ participates in thyroid hormone-associated pathways involved in white adipose tissue browning. Collectively, these findings suggest that LXRs integrate metabolic and endocrine signals to modulate adipocyte thermogenic programs in a context-dependent manner. Consequently, isoform-specific modulation of LXR signaling may represent a potential strategy to regulate adipose tissue metabolism. However, the therapeutic implications of selectively modulating LXR signaling require further investigation, especially in humans.

### 4.5. Farnesoid X Receptor (FXR)

Farnesoid X receptor (FXR), a bile acid-activated nuclear receptor, primarily regulates bile acid metabolism and enterohepatic signaling, but accumulating evidence indicates that bile acid–nuclear receptor pathways can influence systemic energy expenditure and adipose tissue metabolism. Expanding the landscape of non-adrenergic regulators, a recent study investigated a gut–microbiota–adipose axis in which FXR mediates microbiota-derived bile acid signaling [142]. The authors reported that dietary factors influence microbial bile acid metabolism, which in turn modulates FXR activity in adipocyte progenitor cells. This microbiota-driven FXR signaling may contribute to the regulation of beige adipocyte recruitment through mechanisms that are at least partially independent of classical sympathetic activation. Consistent with the emerging role of FXR signaling in adipose regulation, a recent study implicated the FXR-ApoC2 axis as a potential non-adrenergic regulator of thermogenesis [161]. FXR activation was associated with increased browning-related gene expression in white adipose tissue by modulating ApoC2-dependent lipid metabolism. These findings suggest that systemic metabolic signals and nuclear receptor pathways contribute to the regulation of UCP1 expression and thermogenic activity. They also highlight the potential relevance of FXR-associated pathways for metabolic disease intervention. Another recent review discussed the therapeutic potential of fexaramine, a gut-restricted farnesoid X receptor (FXR) agonist, for obesity and metabolic disorders [33]. Preclinical studies summarized in the review suggest that intestine-specific FXR activation improves glucose and lipid homeostasis, insulin sensitivity, and energy expenditure by promoting white adipose tissue browning and enhancing brown and beige adipocyte thermogenesis. The review highlights mechanisms involving interactions with β-adrenergic signaling, TGR5/GLP-1 pathways, and FGF-19/15 induction. These findings suggest that intestinal FXR activation may represent a potential therapeutic strategy for obesity and metabolic disorders by modulating adipose thermogenesis and systemic metabolism.

### 4.6. Thyroid Hormone Receptors (TRs)

Thyroid hormones are pivotal regulators of thermogenesis [180]. Thyroid hormone receptors (TRα and TRβ) bind triiodothyronine (T3) and regulate transcription of genes involved in mitochondrial respiration and UCP1 expression by binding to thyroid response elements within the UCP1 enhancer region. In human BAT, local conversion of thyroxine (T4) to T3 by type II iodothyronine deiodinase (DIO2) enhances intracellular thyroid signaling. TR activation cooperates with cAMP-mediated pathways, amplifying β-adrenergic responses and increasing mitochondrial gene transcription through recruitment of coactivators such as PGC-1α [181]. Thyroid receptor signaling exemplifies cross-talk between endocrine and sympathetic systems and contributes to mitochondrial biogenesis, expression of oxidative phosphorylation genes, and thermogenic responsiveness in brown adipocytes.

Several recent studies support the idea that thyroid hormone (TH) signaling, acting through specific nuclear receptor isoforms, is an important endocrine regulator of thermogenesis. It contributes to multiple stages of thermogenic adipocyte development and function. At the earliest stages of life, TH signaling influences the thermogenic progenitor pool; for instance, maternal activation of thyroid hormone receptor β (TRβ) has been reported to promote long-term enhancement of thermogenic function in offspring [182]. Complementing this developmental priming, locally available T3 signals primarily through the TRα isoform to drive adipocyte progenitor cell proliferation, potentially contributing to brown fat hyperplasia and increased thermogenic potential [162]. Importantly, the requirement for cell-autonomous TH signaling extends well beyond early development and into adulthood. Intact local TH responsiveness within mature brown adipocytes is required to mount an efficient adaptive thermogenic response to environmental challenges [183]. Furthermore, this importance is not restricted to classical brown fat; TH also contributes to efficient induction of thermogenic programs during white fat browning, even when the process is induced by genetic inactivation of the adipogenic repressor Zfp423 [163]. Together, these findings suggest that distinct TR isoforms contribute to thermogenic regulation at different developmental and physiological stages. Through its effects on progenitor expansion and thermogenic gene regulation, TH signaling interacts extensively with sympathetic pathways. It also directly regulates the transcription of thermogenic genes. However, systemic thyroid hormone administration induces cardiovascular side effects, limiting therapeutic application. Tissue-selective TR agonists or approaches designed to minimize off-target effects may represent more clinically viable therapeutic strategies for metabolic disorders.

### 4.7. Retinoid Receptors (RARs and RXRs)

Retinoic acid receptors (RARs) and retinoid X receptors (RXRs) regulate adipocyte differentiation and thermogenic gene expression [179]. RXR functions as a heterodimerization partner for multiple nuclear receptors, including PPARs, LXRs, RARs, and TRs. However, retinoid effects in human BAT are complex and dose-dependent, with potential adverse effects on systemic metabolism. In the pursuit of clinically viable non-adrenergic targets, it is important to consider species-specific translational challenges. An influential study highlighted this by demonstrating that retinoic acid exerts divergent effects on UCP1 expression when comparing mouse and human adipocytes [164]. While retinoid signaling promotes thermogenic programs in murine models, its regulatory impact appears to differ substantially in human adipocytes. This observation highlights the importance of utilizing human-derived experimental models when evaluating retinoid pathways and other alternative receptor signaling networks for therapeutic modulation of adipose browning. Building upon the pharmacological targeting of retinoid networks, a recent study reported that the RARβ agonist adapalene acts as a potential inducer of adipocyte browning through non-classical signaling pathways [165]. Adapalene promoted white adipocyte browning and mitochondrial biogenesis involving activation of the RARβ–p38 MAPK–ATF2 signaling pathway. By identifying a candidate signaling mechanism, the study suggests the potential utility of repurposing existing clinical compounds to modulate thermogenic pathways and metabolic gene programs.

In support of pharmacological modulation of thermogenic pathways, another study identified the RXR agonist bexarotene as an inducer of brown adipogenic reprogramming [167]. Through high-throughput screening, this study demonstrated that pharmacological RXR activation promoted conversion of committed myoblasts into brown adipocyte-like cells expressing thermogenic markers. This chemically induced reprogramming initiated browning-related pathways partially independent of classical adipogenic regulators, suggesting that selective pharmacological activation of nuclear receptors can modulate thermogenic capacity in vivo. In addition, a recent study reported that RXRγ physically and functionally interacts with PPARγ to promote thermogenic differentiation programs during adipose tissue differentiation [166]. This mechanistic insight suggests that specific nuclear receptor heterodimers function as regulators of differentiation-associated thermogenic responses. Collectively, these findings indicate that targeting the RAR/RXR transcriptional complex may provide a potentially selective approach to modulating brown fat activity through mechanisms that may complement sympathetic signaling pathways.

### 4.8. Estrogen-Related Receptors (ERRs)

Estrogen-related receptors (ERRs), particularly ERRα and ERRγ, are orphan nuclear receptors that play important roles in regulating mitochondrial oxidative metabolism [184]. Although no endogenous ligand has been definitively identified, ERR activity is enhanced through interaction with the coactivator PGC-1α, a key regulator of thermogenic gene expression. The ERR–PGC-1α transcriptional complex promotes the expression of genes involved in mitochondrial biogenesis, oxidative phosphorylation, and fatty acid oxidation, thereby supporting the high oxidative capacity associated with brown adipocyte thermogenesis. A recent study established a transcriptional link between ERR signaling and GPR3-associated thermogenic regulation [168]. The study reported that cold exposure activates ERRα and ERRγ-dependent transcriptional programs to increase GPR3 expression, a G-protein coupled receptor that promotes cyclic AMP production in a ligand-independent manner. By positioning ERRs upstream of GPR3 regulation, this study suggests a ligand-independent regulatory mechanism that may support oxidative and thermogenic gene programs in brown adipocytes beyond canonical β-adrenergic signaling. In addition to transcriptional regulation, a recent study reported that the protease SENP2 contributes to regulation of thermogenic gene expression through modulation of ERRα activity [169]. SENP2 directly deSUMOylates ERRα, thereby enhancing ERRα-associated transcriptional activity and increasing UCP1 expression in brown adipocytes. This post-translational regulatory mechanism suggests that modulation of specific nuclear receptor signaling pathways may influence thermogenic gene expression through mechanisms that complement sympathetic signaling. Thus, ERRs coordinate metabolic transcriptional networks that support oxidative metabolism in thermogenic adipocytes and serve as important regulators linking mitochondrial metabolism to thermogenic function.

### 4.9. Androgen Receptors, Glucocorticoid Receptors, and Mineralocorticoid Receptors (ARs, GRs, MRs)

Within the nuclear receptor network, the steroid hormone receptors—specifically the androgen receptors (ARs), glucocorticoid receptors (GRs), and mineralocorticoid receptors (MRs)—constitute an important class of endocrine regulators that modulate brown and beige adipocyte function, often through inhibitory effects on thermogenic programs. While GR classically represses UCP1 expression to conserve energy during physiological stress, both AR and MR signaling have similarly been shown to suppress thermogenic reprogramming and promote adipose tissue whitening. Collectively, these three receptors function as endogenous modulators that can restrain thermogenic activity and energy expenditure, highlighting that targeted steroid receptor antagonism could represent a potential pharmacological strategy to enhance brown fat activity.

Adding a crucial layer of sex-specific endocrine regulation, a study identified AR as a negative regulator of beige adipogenesis [170]. AR activation directly suppresses the expression of PRDM16, a key transcriptional regulator of thermogenic cell fate. This finding highlights how non-adrenergic steroid hormone signaling can suppress the browning of white fat, providing a potential mechanism that may contribute to sex-dependent differences in adipose tissue plasticity.

In addition to the role of AR signaling in thermogenesis, a study detailed the inhibitory role of the glucocorticoid receptor (GR) in white adipose tissue beiging [172]. The authors outlined how elevated glucocorticoid signaling represses UCP1 expression and mitochondrial biogenesis, suppressing adipocyte browning-associated gene expression. This positions GR as an important endocrine regulator of thermogenic adaptation that contributes to energy conservation during physiological stress, suggesting that modulation of this pathway may influence thermogenic responses and adipose tissue remodeling. Supporting the role of glucocorticoid signaling in thermogenic regulation, the selective glucocorticoid receptor antagonist CORT125281 increased BAT activity in male mice [173]. This targeted pharmacological blockade enhanced BAT activity and altered systemic lipid distribution, highlighting GR antagonism as a potential therapeutic strategy.

MR, traditionally known for regulating electrolyte balance, has emerged as an important modulator of adipocyte biology [42]. MR activation promotes oxidative stress and inflammation in adipose tissue, stimulates white adipocyte differentiation, and suppresses brown adipocyte development and thermogenic activity in mice [174]. Consistently, pharmacological MR antagonism has been reported to improve adipose tissue function and enhance BAT thermogenic activity, suggesting that MR signaling may represent a potential therapeutic target for obesity and metabolic disorders.

### 4.10. Therapeutic Potential of Targeting Nuclear Receptors in BAT

NRs represent an important class of transcriptional regulators that integrate hormonal, nutritional, and metabolic cues to control BAT function. Several NRs, including PPARγ, PPARα/δ, and TRs, directly promote thermogenic gene expression and mitochondrial activity, supporting their potential as therapeutic targets to enhance energy expenditure. Pharmacological activation of these receptors has demonstrated beneficial metabolic effects in experimental models; however, their pleiotropic roles—particularly the adipogenic effects of PPARγ—pose challenges for selective therapeutic application [185]. Conversely, NRs such as LXRs, GR, MR, and AR have generally been associated with suppression of thermogenic programs by promoting lipogenesis, stress signaling, or inflammatory pathways. Inhibition or modulation of these pathways may therefore represent an alternative strategy to enhance BAT activity by reducing inhibitory signaling, particularly in obesity where these signaling axes are often dysregulated. For instance, MR antagonism and selective GR modulation have been associated with improved metabolic phenotypes and enhanced thermogenic responses [42,173].

Additional regulatory complexity is introduced by RXR, which functions as a central heterodimerization partner, and ERRs, which coordinate mitochondrial biogenesis through coactivation with PGC-1α. Targeting these integrative nodes may influence multiple aspects of BAT metabolic regulation, although achieving tissue specificity remains a key challenge. Notably, many of these NRs are expressed in human BAT, and their activity has been reported to be altered in obesity, suggesting potential translational relevance. Future therapeutic strategies will likely require selective modulators that enhance BAT-selective thermogenic activation without eliciting adverse systemic effects, potentially through tissue-targeted delivery or pathway-biased signaling approaches.

## 5. Conclusions and Future Directions

**Figure 2:**
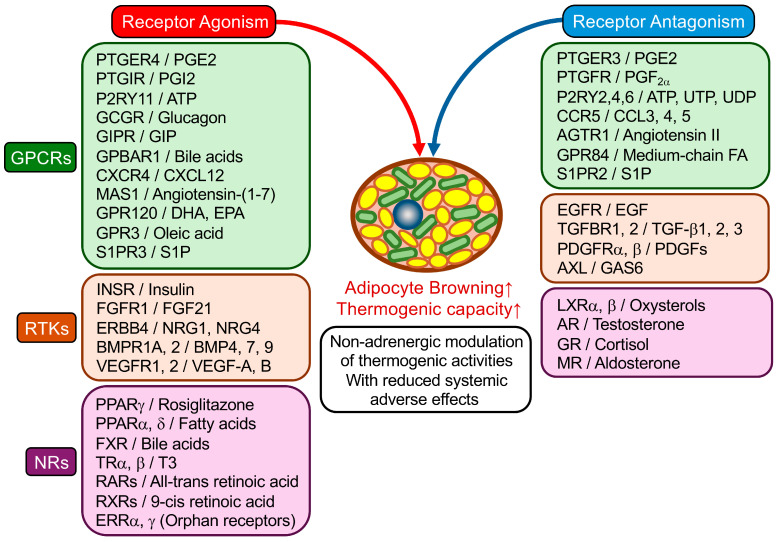
**Pharmacological strategies for modulating thermogenic activity in human brown adipocytes.** Agonism or antagonism of various non-adrenergic receptor pathways has been implicated in the regulation of adipocyte browning and thermogenic capacity in human brown adipocytes. Selective modulation of these receptor pathways may influence thermogenic gene expression programs with potentially reduced systemic adverse effects. Further studies are needed to clarify mechanistic links between findings from preclinical models and observations in humans. In addition, the development of selective agonists, antagonists, or allosteric modulators may facilitate the evaluation of these pathways as therapeutic targets for regulating adipocyte browning and thermogenic capacity of human brown adipocytes. Combinatorial modulation of receptor signaling pathways represents an area of active investigation and may provide an additional strategy for modulating brown adipocyte function in humans.

Non-adrenergic signaling pathways, including GPCRs, RTKs, and NRs, comprise interconnected regulatory pathways that control human brown adipocyte function beyond classical β-adrenergic stimulation (Figure 2). Many of these pathways converge on processes involved in cAMP signaling, calcium dynamics, mitochondrial activity, and transcriptional control of thermogenic gene programs, thereby fine-tuning BAT activation in response to diverse hormonal, metabolic, and inflammatory cues [15]. Importantly, accumulating evidence indicates that many of these receptors are expressed in human BAT and some have been reported to exhibit altered expression or activity in obesity, supporting their potential physiological and translational relevance, as described above.

Targeting alternative receptor signaling offers a promising avenue to overcome some limitations of β-adrenergic-based therapies, which have shown restricted efficacy and safety concerns in humans. However, the functional outcomes of these pathways are highly context-dependent, with some receptors promoting thermogenesis while others exert inhibitory or modulatory effects. This highlights the need for a more nuanced understanding of receptor-specific signaling, ligand bias, and cell-type-specific actions within the heterogeneous cellular environment of BAT.

Future studies should prioritize the identification of human-specific regulatory mechanisms, including receptors that exhibit species-specific expression, signaling, or functional properties, as well as the integration of multi-omics and single-cell approaches to better characterize cellular heterogeneity. Systematic validation using human primary cells, organoid systems, and in vivo imaging will be essential to determine whether these receptor signaling pathways play functionally relevant roles in human BAT biology and whether they represent potential targets for metabolic intervention. In addition, developing tissue-targeted or pathway-selective modulators will be critical to enhance thermogenic activity while minimizing systemic side effects. Ultimately, a comprehensive understanding of non-adrenergic receptor signaling in human BAT may facilitate the development of next-generation therapeutics for obesity and related metabolic disease.

## Figures and Tables

**Table 1:** **Non-Adrenergic GPCRs Associated with Human Brown Adipocyte Regulation**.

Receptor	Major Ligand(s)	G- Protein	Effect on Thermogenic Function	Potential Relevance to Human BAT *	Ref.
PTGER3 (EP3)	Prostaglandin E2 (PGE2)	Gi	May suppress cAMP-dependent thermogenic signaling, potentially reducing UCP1 expression and mitochondrial activity; acts as a negative regulator of brown and beige adipocyte thermogenesis	Expressed in human BAT; EP3 signaling is associated with reduced energy expenditure and may contribute to obesity by suppressing thermogenic capacity;	[59,60]
PTGER4 (EP4)	Prostaglandin E2 (PGE2)	Gs	Stimulates cAMP production and CREB-dependent transcription, potentially promoting UCP1 expression and mitochondrial biogenesis with additional anti-inflammatory signaling properties.	Expressed in human primary brown adipocytes; obesity-associated inflammation and prostaglandin imbalance may modify EP4 signaling, potentially influencing BAT activation and metabolic flexibility.	[61,62]
PTGIR (IP)	Prostacyclin (PGI2)	Gs	Activates cAMP-dependent signaling and is particularly linked to adipocyte differentiation and browning programs, potentially enhancing mitochondrial capacity and thermogenic gene expression.	Expressed in human primary brown adipocytes; impaired prostacyclin signaling in obesity may reduce browning capacity and limit adaptive thermogenesis	[63,64]
PTGFR (FP)	Prostaglandin F_2_α (PGF_2_α)	Gq	Activates Gq–Ca^2+^ signaling and downstream pathways such as PKC, which may counteract cAMP-driven thermogenesis and modulate mitochondrial dynamics in a context-dependent manner.	Expressed in human BAT; elevated PGF_2_α signaling in obesity may contribute to suppressed thermogenic activity and altered adipose tissue remodeling.	[65,66]
P2RY2/ P2RY4/ P2RY6	ATP, UTP (P2RY2/4), UDP (P2RY6)	Gq	Activation of these receptors primarily induces Gq–Ca^2+^ signaling, integrating extracellular nucleotide stress signals to influence mitochondrial dynamics and fine-tune thermogenic gene expression in brown adipocytes.	Expressed in human primary brown adipocytes; obesity-associated alterations in extracellular nucleotide release and inflammation may dysregulate their signaling, potentially impairing BAT metabolic adaptation.	[49,67]
P2RY11	ATP	Gs and Gq	Uniquely exhibits dual Gs/Gq coupling, enabling simultaneous activation of cAMP and Ca^2+^ pathways, which may synergistically affect mitochondrial activity and thermogenic gene expression in brown adipocytes.	Expressed in human primary brown adipocytes, but absent in rodents; obesity-related changes in purinergic signaling may alter its activity, with potential implications for human-specific regulation of BAT thermogenesis.	[68,69]
GCGR	Glucagon	Gs	Activates Gs–cAMP signaling to promote lipolysis and increase substrate availability, potentially supporting mitochondrial respiration and thermogenic programming in brown adipocytes.	Expressed in human primary brown adipocytes; obesity-associated alterations in glucagon sensitivity and signaling may influence BAT activation and systemic energy expenditure	[70,71]
GIPR	Glucose-dependent insulinotropic polypeptide (GIP), long-acting GIP analogs	Gs	GIPR activation has been reported to affect lipid uptake, lipid handling, and fatty acid oxidation in brown adipose tissue, suggesting a context-dependent role in supporting thermogenic metabolism; however, its direct effect on UCP1-driven thermogenesis remains less clearly defined	Expressed in human BAT; GIPR signaling contributes to systemic metabolic regulation; recent studies indicate that pharmacological activation (e.g., tirzepatide) modulates adipocyte nutrient metabolism and may improve metabolic function partly via BAT	[72,73]
GPBAR1 (TGR5)	Primary and secondary bile acids, bile acid derivatives	Gs	Acts as a bile acid sensor that activates cAMP signaling, promoting UCP1 expression, mitochondrial respiration, and energy dissipation in brown adipocytes.	Expressed in human BAT; obesity-associated changes in bile acid composition and signaling may influence GPBAR1 activity, thereby affecting BAT-mediated energy expenditure.	[73,74]
CCR5	CCL3, CCL4, CCL5	Gi	Activates Gi-dependent chemokine signaling linked to immune cell recruitment and inflammatory cascades, which may suppress cAMP signaling and inhibit thermogenic gene expression in brown adipocytes.	Expressed in human adipocytes; obesity-associated chronic inflammation enhances CCR5 signaling, contributing to immune infiltration and impaired thermogenic capacity.	[75]
CXCR4	CXCL12 (SDF-1)	Gi, p38 and ERK signaling	CXCR4 signaling has been reported to support thermogenic gene expression and mitochondrial function; adipocyte-specific CXCR4 deficiency impairs BAT thermoregulation and reduces thermogenic gene expression	Expressed in human adipocytes; CXCR4 signaling potentially protects against diet-induced obesity, preserves insulin sensitivity, and maintains brown adipocyte activity during metabolic stress	[76,77]
AGTR1 (AT1R)	Angiotensin II	Gq	Activates Gq–PLC–Ca^2+^ signaling and downstream oxidative stress and profibrotic pathways, potentially leading to mitochondrial dysfunction and suppression of thermogenic activity.	Expressed in human primary brown adipocytes; upregulated in obesity via local renin–angiotensin system activation, contributing to BAT dysfunction and reduced energy expenditure	[78]
MAS1	Angiotensin- (1–7)	Gi	Counteracts AGTR1 signaling by promoting anti-inflammatory and vasodilatory pathways, which may support mitochondrial function and enhance thermogenic gene expression.	Expressed in human primary brown adipocytes; its protective axis is diminished in obesity, potentially contributing to reduced BAT activity and metabolic inefficiency.	[78,79]
GPR120 (FFAR4)	Long-chain unsaturated fatty acids (omega-3 FA such as DHA, EPA)	Gq and β-arrestin	Acts as a lipid sensor mediating anti-inflammatory and insulin-sensitizing signaling via β-arrestin, which may enhance mitochondrial activity and thermogenic gene expression.	Expressed in human BAT; impaired signaling or loss-of-function variants in obesity are linked to metabolic dysfunction and reduced BAT-mediated energy expenditure.	[80,81]
GPR3	Oleic acid (Constitutively active)	Gs	Exhibits ligand-independent constitutive activity, contributing to basal cAMP levels that sustain UCP1 expression and intrinsic thermogenic programming.	Expressed in human primary brown adipocytes; reduced expression in obesity may contribute to decreased basal thermogenesis and impaired energy balance.	[82,83,84]
GPR84	Medium-chain fatty acids (C9–C14 fatty acids)	Gi	Functions as a nutrient and inflammation-responsive receptor, activating Gi-mediated pro-inflammatory pathways that may suppress cAMP signaling and thermogenic programs.	Expressed in human primary brown adipocytes; upregulated in obesity-associated inflammation, potentially contributing to reduced thermogenic capacity.	[85,86]
S1PR2	S1P	Gi, Gq, G12/13	Preferentially activates RhoA/ROCK signaling, which may lead to cytoskeletal remodeling and inhibition of mitochondrial function and thermogenic gene expression.	Expressed in human primary brown adipocytes; obesity-associated sphingolipid accumulation enhances S1PR2 signaling, potentially contributing to metabolic dysfunction, with inhibition improving metabolic phenotypes.	[87,88]
S1PR3	S1P	Gi, Gq, G12/13	Integrates multiple G-protein pathways to regulate calcium signaling, vascular tone, and inflammatory responses, exerting context-dependent effects on mitochondrial activity and thermogenesis.	Expressed in human primary brown adipocytes; obesity-driven alterations in sphingolipid signaling and inflammation may dysregulate S1PR3 activity and impair BAT function.	[89]

* mechanistic evidence is derived primarily from rodent studies unless otherwise indicated.

**Table 2:** **Receptor Tyrosine Kinases Associated with Human Brown Adipocyte Regulation**.

Receptor	Major Ligand(s)	Downstream Signaling	Effect on Thermogenic Function	Potential Relevance to Human BAT *	Ref.
INSR (Insulin Receptor)	Insulin	PI3K–AKT, MAPK, mTOR	Coordinates glucose uptake and anabolic metabolism, providing substrates for mitochondrial respiration and supporting aspects of brown adipocyte function.	Expressed in human brown adipocytes; insulin resistance in obesity impairs glucose utilization and metabolic flexibility, which may contribute to reduced thermogenic capacity.	[116]
FGFR1	FGF21 (β-Klotho dependent), FGF9, FGF6	MAPK/ERK, PI3K–AKT, PLCγ	Functions as an important endocrine signaling modulator, where FGF21–FGFR1 signaling enhances UCP1 expression, mitochondrial biogenesis, and browning of adipocytes.	Expressed in human brown adipocytes; pharmacological activation has been shown to improve metabolic parameters in preclinical and some clinical studies, although obesity is associated with FGF21 resistance.	[32]
EGFR (ERBB1)	EGF, TGF-α, Amphiregulin	MAPK/ERK, PI3K–AKT	Primarily regulates adipose tissue remodeling and inflammatory signaling rather than directly promoting thermogenesis, with potential inhibitory effects on adipocyte differentiation under sustained activation.	Expressed in adipose tissue-resident immune cells, including those within human BAT; obesity-associated EGFR activation in immune cells contributes to inflammation and insulin resistance.	[117]
ERBB4	Neuregulins (NRG1, NRG4)	PI3K–AKT, MAPK/ERK	Mediates NRG4-dependent signaling associated with enhanced mitochondrial function, lipid utilization, and thermogenic gene expression in brown adipocytes.	Expressed in human BAT; BAT-derived NRG4 acts as an endocrine factor protecting against obesity-associated metabolic dysfunction and hepatic steatosis in mouse models.	[118,119]
TGFBR1 (ALK5)/TGFBR2	TGF-β1, TGF-β2, TGF-β3	SMAD2/3 signaling, MAPK	Functions as a negative regulator of thermogenesis by inhibiting brown/beige adipocyte differentiation and repressing UCP1 expression.	Expressed in human BAT; TGF-β signaling is elevated in obesity, promoting fibrosis and suppressing BAT thermogenic capacity.	[120,121]
BMPR1A (ALK3)/BMPR2	BMP4, BMP7, BMP9	SMAD1/5/8 signaling, p38 MAPK	Regulates lineage commitment toward brown/beige adipocytes and activates thermogenic gene programs in a ligand- and context-dependent manner.	Expressed in human BAT; BMP signaling plays an important role in adipose development, and its dysregulation may impair thermogenic remodeling in obesity.	[122,123]
PDGFRα	PDGF-AA, PDGF-AB, PDGF-CC	PI3K–AKT, MAPK/ERK, JAK–STAT	Regulates adipocyte progenitor proliferation and lineage specification; increased signaling is associated with fibrotic remodeling and reduced thermogenic differentiation.	Expressed in stromal progenitors within human BAT; obesity-associated activation is associated with fibrosis and suppresses brown/beige adipocyte formation.	[124,125]
PDGFRβ	PDGF-AB, PDGF-BB, PDGF-DD	PI3K–AKT, MAPK/ERK, JAK–STAT	Controls vascular and perivascular cell function, contributing to regulation of adipose microenvironment that may influence thermogenic function.	Expressed in human BAT-associated vasculature; increased signaling in obesity contributes to tissue remodeling and metabolic dysfunction.	[126,127]
VEGFR1 (FLT1)/VEGFR2 (KDR)	VEGF-A, VEGF-B, PlGF	PI3K–AKT, MAPK/ERK	Primarily regulates angiogenesis and vascular permeability, which may indirectly support thermogenesis by facilitating oxygen and nutrient delivery to metabolically active BAT.	Expressed in human BAT vasculature; impaired VEGF signaling in obesity disrupts vascularization, limiting BAT expansion and thermogenic function.	[128,129]
AXL (TAM receptor family)	Growth arrest-specific 6 (GAS6)	PI3K–AKT, MAPK/ERK, JAK–STAT	AXL signaling has been reported to suppress UCP1 expression and mitochondrial oxidative metabolism in brown adipocytes.	Expressed in preadipocytes within human BAT; AXL deficiency enhances BAT thermogenic activity and energy expenditure and protects against diet-induced obesity in mouse models; AXL signaling has been reported to be upregulated in obesity-associated inflammatory states.	[130]

* mechanistic evidence is derived primarily from rodent studies unless otherwise indicated.

**Table 3:** **Nuclear Receptors Associated with Human Brown Adipocyte Regulation**.

Receptor	Major Ligand(s)	Downstream Signaling	Effect on Thermogenic Function	Potential Relevance to Human BAT *	Ref.
PPARγ (NR1C3)	Fatty acids, prostaglandin derivatives, thiazolidinediones	PPARγ–RXR heterodimer, recruitment of PGC-1α	A key regulator of adipogenesis that contributes to the establishment of brown adipocyte identity and UCP1 expression in cooperation with PGC-1α.	Expressed in human brown adipocytes; a pharmacological target in diabetes, but excessive activation promotes adiposity, although it supports aspects of thermogenic programming.	[37,155]
PPARα (NR1C1)	Long-chain fatty acids, eicosanoids, fibrates	PPARα–RXR transcriptional regulation of fatty acid oxidation (FAO) genes	Promotes mitochondrial fatty acid β-oxidation and supports fatty acid utilization and oxidative metabolism in thermogenic adipocytes.	Expressed in human brown adipocytes; activation improves lipid metabolism and may enhance BAT oxidative capacity in metabolic disease.	[156,157]
PPARδ (NR1C2)	Polyunsaturated fatty acids	PPARδ–RXR transcriptional regulation of oxidative metabolism genes	Supports mitochondrial biogenesis and oxidative metabolism, thereby contributing to thermogenic function and energy expenditure.	Expressed in human BAT; has been associated with resistance to obesity by promoting lipid utilization and increasing whole-body energy expenditure in a mouse model.	[158]
LXRα/LXRβ (NR1H3/NR1H2)	Oxysterols	LXR–RXR transcriptional regulation of lipogenic genes	Favors lipid storage pathways and has been associated with suppression of UCP1 expression and thermogenic gene programs.	Expressed in human BAT; activation is associated with dyslipidemia and may impair BAT-mediated energy dissipation in obesity.	[159,160]
FXR (NR1H4)	Bile acids (e.g., chenodeoxycholic acid)	FXR–RXR transcriptional regulation of metabolic genes	Primarily modulates systemic energy metabolism via bile acid signaling, with context-dependent effects on thermogenesis.	Expressed in human BAT; altered bile acid–FXR signaling in obesity influences metabolic homeostasis and energy balance.	[142,161]
TRα/ TRβ (NR1A1/ NR1A2)	Triiodothyronine (T3), thyroxine (T4)	TR–RXR transcriptional regulation of thermogenic and mitochondrial genes	Promotes UCP1 expression, mitochondrial biogenesis, and oxidative phosphorylation, supporting thermogenic function.	Expressed in human brown adipocytes; play an important role in endocrine regulation of BAT activation and systemic energy expenditure.	[162,163]
RARα/ RARβ/ RARγ (NR1B1/ NR1B2/NR1B3)	All-trans retinoic acid (ATRA)	RAR–RXR transcriptional regulation via retinoic acid-responsive metabolic genes	Regulates UCP1 expression and mitochondrial oxidative metabolism, contributing to thermogenic gene activation.	Expressed in human brown adipocytes; retinoid signaling regulates adipocyte differentiation and energy expenditure, with potential implications for obesity-related metabolic regulation.	[164,165]
RXRα/ RXRβ/ RXRγ (NR2B1/ NR2B2/NR2B3)	9-cis retinoic acid	Heterodimerization with multiple nuclear receptors (PPARs, LXRs, RARs, TRs)	Functions as a transcriptional integrator through heterodimerization with multiple nuclear receptors, thereby contributing to coordinated regulation of thermogenic and metabolic gene networks.	Expressed in human brown adipocytes; participates in multiple nuclear receptor pathways involved in adipocyte function and systemic metabolism.	[166,167]
ERRα ERRγ (NR3B1/ NR3B3)	Orphan receptors	ERR–PGC-1α transcriptional regulation of mitochondrial biogenesis and oxidative genes	Important regulators of mitochondrial biogenesis, oxidative metabolism, and respiratory capacity in brown adipocytes.	Expressed in human BAT; they have been implicated in adaptive thermogenesis and maintenance of oxidative capacity.	[168,169]
AR (NR3C4)	Testosterone, dihydrotestosterone	AR homodimer transcriptional regulation of androgen-responsive metabolic signaling pathways	Potentially suppresses thermogenic gene expression and mitochondrial activity, which may limit thermogenic adipocyte differentiation and activity.	Expressed in human BAT; contributes to sex differences in adipose distribution and metabolic phenotype.	[170,171]
GR (NR3C1)	Cortisol, corticosterone	GRE-dependent transcription regulating stress-responsive metabolic gene programs	Can inhibit UCP1 expression and thermogenic activation, particularly under chronic exposure conditions.	Expressed in human BAT; chronic glucocorticoid exposure has been associated with reduced BAT activity and metabolic dysfunction in rats and mice.	[172,173]
MR (NR3C2)	Aldosterone, corticosterone	MRE-dependent transcription regulating mineralocorticoid-responsive metabolic gene programs	Has been associated with suppression of thermogenic gene expression and adipose inflammation, although context-dependent effects on BAT function have also been reported.	Expressed in human BAT; MR overactivation in obesity has been associated with metabolic dysfunction, while antagonism has been reported to improve adipose browning and metabolic phenotypes in mice.	[174,175]

* mechanistic evidence is derived primarily from rodent studies unless otherwise indicated.

## Data Availability

No new data were created or analyzed in this study. Data sharing is not applicable to this article.

## References

[1] Blüher M. (2019). Obesity: Global Epidemiology and Pathogenesis. Nat. Rev. Endocrinol..

[2] Murray C.J., Aravkin A.Y., Zheng P., Abbafati C., Abbas K.M., Abbasi-Kangevari M., Abd-Allah F., Abdelalim A., Abdollahi M., Abdollahpour I. (2020). Global Burden of 87 Risk Factors in 204 Countries and Territories, 1990–2019: A Systematic Analysis for the Global Burden of Disease Study 2019. Lancet.

[3] Clayton B.E., Guertin D.A. (2026). Brown Adipose Tissue Remains Hot. Nat. Rev. Endocrinol..

[4] Karanfil A.S., Louis F., Matsusaki M. (2026). Brown Adipose Tissue Engineering: Advances, Challenges, and Future Directions. Trends Biotechnol..

[5] Saito M., Okamatsu-Ogura Y., Matsushita M., Watanabe K., Yoneshiro T., Nio-Kobayashi J., Iwanaga T., Miyagawa M., Kameya T., Nakada K. (2009). High Incidence of Metabolically Active Brown Adipose Tissue in Healthy Adult Humans: Effects of Cold Exposure and Adiposity. Diabetes.

[6] Cypess A.M., Lehman S., Williams G., Tal I., Rodman D., Goldfine A.B., Kuo F.C., Palmer E.L., Tseng Y.-H., Doria A. (2009). Identification and Importance of Brown Adipose Tissue in Adult Humans. N. Engl. J. Med..

[7] Au-Yong I.T.H., Thorn N., Ganatra R., Perkins A.C., Symonds M.E. (2009). Brown Adipose Tissue and Seasonal Variation in Humans. Diabetes.

[8] Bartelt A., Bruns O.T., Reimer R., Hohenberg H., Ittrich H., Peldschus K., Kaul M.G., Tromsdorf U.I., Weller H., Waurisch C. (2011). Brown Adipose Tissue Activity Controls Triglyceride Clearance. Nat. Med..

[9] Stanford K.I., Middelbeek R.J.W., Townsend K.L., An D., Nygaard E.B., Hitchcox K.M., Markan K.R., Nakano K., Hirshman M.F., Tseng Y.-H. (2013). Brown Adipose Tissue Regulates Glucose Homeostasis and Insulin Sensitivity. J. Clin. Investig..

[10] Cannon B., de Jong J.M.A., Fischer A.W., Nedergaard J., Petrovic N. (2020). Human Brown Adipose Tissue: Classical Brown Rather than Brite/Beige?. Exp. Physiol..

[11] Blondin D.P., Nielsen S., Kuipers E.N., Severinsen M.C., Jensen V.H., Miard S., Jespersen N.Z., Kooijman S., Boon M.R., Fortin M. (2020). Human Brown Adipocyte Thermogenesis Is Driven by B2-AR Stimulation. Cell Metab..

[12] Becher T., Palanisamy S., Kramer D.J., Eljalby M., Marx S.J., Wibmer A.G., Butler S.D., Jiang C.S., Vaughan R., Schöder H. (2021). Brown Adipose Tissue Is Associated with Cardiometabolic Health. Nat. Med..

[13] Nedergaard J., Cannon B. (2010). The Changed Metabolic World with Human Brown Adipose Tissue: Therapeutic Visions. Cell Metab..

[14] Takeda Y., Harada Y., Yoshikawa T., Dai P. (2023). Mitochondrial Energy Metabolism in the Regulation of Thermogenic Brown Fats and Human Metabolic Diseases. Int. J. Mol. Sci..

[15] Cohen P., Kajimura S. (2021). The Cellular and Functional Complexity of Thermogenic Fat. Nat. Rev. Mol. Cell Biol..

[16] Roesler A., Kazak L. (2020). UCP1-Independent Thermogenesis. Biochem. J..

[17] Ikeda K., Yamada T. (2022). Adipose Tissue Thermogenesis by Calcium Futile Cycling. J. Biochem..

[18] Rahbani J.F., Roesler A., Hussain M.F., Samborska B., Dykstra C.B., Tsai L., Jedrychowski M.P., Vergnes L., Reue K., Spiegelman B.M. (2021). Creatine Kinase B Controls Futile Creatine Cycling in Thermogenic Fat. Nature.

[19] Sun Y., Rahbani J.F., Jedrychowski M.P., Riley C.L., Vidoni S., Bogoslavski D., Hu B., Dumesic P.A., Zeng X., Wang A.B. (2021). Mitochondrial TNAP Controls Thermogenesis by Hydrolysis of Phosphocreatine. Nature.

[20] Bunk J., Hussain M.F., Delgado-Martin M., Samborska B., Ersin M., Shaw A., Rahbani J.F., Kazak L. (2025). The Futile Creatine Cycle Powers UCP1-Independent Thermogenesis in Classical BAT. Nat. Commun..

[21] Sharma A.K., Khandelwal R., Zurkovic J., Long F., Wang T., Dewal R.S., Wu C., Ghosh A., Manuel K., Othman A. (2026). DGAT-Driven Futile Lipid Cycling Has a Pronounced, yet Concealed, Thermogenic Function. Cell Metab..

[22] Liu X., He A., Lu D., Hu D., Tan M., Abere A., Goodarzi P., Ahmad B., Kleiboeker B., Finck B.N. (2025). Peroxisomal Metabolism of Branched Fatty Acids Regulates Energy Homeostasis. Nature.

[23] Blondin D.P. (2026). Brown Adipose Tissue Thermogenesis. Physiology.

[24] Wu J., Boström P., Sparks L.M., Ye L., Choi J.H., Giang A.-H., Khandekar M., Virtanen K.A., Nuutila P., Schaart G. (2012). Beige Adipocytes Are a Distinct Type of Thermogenic Fat Cell in Mouse and Human. Cell.

[25] Yoneshiro T., Aita S., Matsushita M., Kayahara T., Kameya T., Kawai Y., Iwanaga T., Saito M. (2013). Recruited Brown Adipose Tissue as an Antiobesity Agent in Humans. J. Clin. Investig..

[26] Chondronikola M., Volpi E., Børsheim E., Porter C., Annamalai P., Enerbäck S., Lidell M.E., Saraf M.K., Labbe S.M., Hurren N.M. (2014). Brown Adipose Tissue Improves Whole-Body Glucose Homeostasis and Insulin Sensitivity in Humans. Diabetes.

[27] Boychenko S., Egorova V.S., Brovin A., Egorov A.D. (2024). White-to-Beige and Back: Adipocyte Conversion and Transcriptional Reprogramming. Pharmaceuticals.

[28] Brown Z., Yoneshiro T. (2024). Brown Fat and Metabolic Health: The Diverse Functions of Dietary Components. Endocrinol. Metab..

[29] Tabei S., Chamorro R., Meyhöfer S.M., Wilms B. (2024). Metabolic Effects of Brown Adipose Tissue Activity Due to Cold Exposure in Humans: A Systematic Review and Meta-Analysis of RCTs and Non-RCTs. Biomedicines.

[30] Al Mahri S., Okla M., Rashid M., Malik S.S., Iqbal J., Al Ibrahim M., Dairi G., Mahmood A., Muthurangan M., Yaqinuddin A. (2023). Profiling of G-Protein Coupled Receptors in Adipose Tissue and Differentiating Adipocytes Offers a Translational Resource for Obesity/Metabolic Research. Cells.

[31] Malfacini D., Pfeifer A. (2023). GPCR in Adipose Tissue Function—Focus on Lipolysis. Biomedicines.

[32] Li C.-X., Tan C.-F., Zhang Q.-M., Qin L.-G., Cao C.-Y., Huang X.-F. (2026). FGF21 Promotes Thermogenesis by Browning Thermogenic Adipose Tissue during Cold Exposure. Ann. Nutr. Metab..

[33] Dąbrowska A.M., Dudka J. (2025). Fexaramine as the Intestine-Specific Farnesoid X Receptor Agonist: A Promising Agent to Treat Obesity and Metabolic Disorders. Drug Discov. Today.

[34] Li Z., Chu H., Yang L. (2025). White Adipose Tissue Browning and Peroxisome Proliferator Activated Receptors in MASLD. Front. Endocrinol..

[35] Manning B.D., Toker A. (2017). AKT/PKB Signaling: Navigating the Network. Cell.

[36] Hossain M.A., Poojari A., Rabiee A. (2026). Thermogenesis in Adipose Tissue: Adrenergic and Non-Adrenergic Pathways. Cells.

[37] Seale P., Bjork B., Yang W., Kajimura S., Chin S., Kuang S., Scimè A., Devarakonda S., Conroe H.M., Erdjument-Bromage H. (2008). PRDM16 Controls a Brown Fat/Skeletal Muscle Switch. Nature.

[38] Jeon Y.G., Kim S.W., Kim J.B. (2024). Decoding Temporal Thermogenesis: Coregulator Selectivity and Transcriptional Control in Brown and Beige Adipocytes. Adipocyte.

[39] Lyons A., Coleman M., Riis S., Favre C., O’Flanagan C.H., Zhdanov A.V., Papkovsky D.B., Hursting S.D., O’Connor R. (2017). Insulin-like Growth Factor 1 Signaling Is Essential for Mitochondrial Biogenesis and Mitophagy in Cancer Cells. J. Biol. Chem..

[40] Luo Z., Yao J., Wang Z., Xu J. (2023). Mitochondria in Endothelial Cells Angiogenesis and Function: Current Understanding and Future Perspectives. J. Transl. Med..

[41] Herz C.T., Kiefer F.W. (2020). The Transcriptional Role of Vitamin A and the Retinoid Axis in Brown Fat Function. Front. Endocrinol..

[42] Armani A., Gorini S., Feraco A., Mammi C., Bellucci E., Caprio M. (2026). Targeting Mineralocorticoid Receptors to Treat Metabolic Diseases via the Adipocyte. Endocrinology.

[43] Samanta S., Bagchi D., Bagchi M. (2024). Physiological and Metabolic Functions of the B3-Adrenergic Receptor and an Approach to Therapeutic Achievements. J. Physiol. Biochem..

[44] Dwaib H.S., Michel M.C. (2023). Is the B3-Adrenoceptor a Valid Target for the Treatment of Obesity and/or Type 2 Diabetes?. Biomolecules.

[45] Straat M.E., Hoekx C.A., van Velden F.H.P., Pereira Arias-Bouda L.M., Dumont L., Blondin D.P., Boon M.R., Martinez-Tellez B., Rensen P.C.N. (2023). Stimulation of the Beta-2-Adrenergic Receptor with Salbutamol Activates Human Brown Adipose Tissue. Cell Rep. Med..

[46] Ishida Y., Matsushita M., Yoneshiro T., Saito M., Fuse S., Hamaoka T., Kuroiwa M., Tanaka R., Kurosawa Y., Nishimura T. (2024). Genetic Evidence for Involvement of B2-Adrenergic Receptor in Brown Adipose Tissue Thermogenesis in Humans. Int. J. Obes..

[47] Gnad T., Navarro G., Lahesmaa M., Reverte-Salisa L., Copperi F., Cordomi A., Naumann J., Hochhäuser A., Haufs-Brusberg S., Wenzel D. (2020). Adenosine/A2B Receptor Signaling Ameliorates the Effects of Aging and Counteracts Obesity. Cell Metab..

[48] Verma N., Perie L., Silvestro M., Verma A., Cronstein B.N., Ramkhelawon B., Mueller E. (2025). Metabolic Dysfunction in Mice with Adipocyte-Specific Ablation of the Adenosine A2A Receptor. J. Biol. Chem..

[49] Wang D., Zhou J. (2023). Purinergic Receptor: A Crucial Regulator of Adipose Tissue Functions. Purinergic Signal..

[50] Cypess A.M., Chen Y.-C., Sze C., Wang K., English J., Chan O., Holman A.R., Tal I., Palmer M.R., Kolodny G.M. (2012). Cold but Not Sympathomimetics Activates Human Brown Adipose Tissue in Vivo. Proc. Natl. Acad. Sci. USA.

[51] Yoneshiro T., Matsushita M., Sakai J., Saito M. (2025). Brown Fat Thermogenesis and Cold Adaptation in Humans. J. Physiol. Anthropol..

[52] Michel M.C., Onaran O. (2026). Biased Agonism at β-Adrenoceptor Subtypes: A Drug Development Perspective. Handb. Exp. Pharmacol..

[53] Carpentier A.C., Blondin D.P. (2023). Human Brown Adipose Tissue Is Not Enough to Combat Cardiometabolic Diseases. J. Clin. Investig..

[54] Dąbrowska A.M., Dudka J. (2023). Mirabegron, a Selective B3-Adrenergic Receptor Agonist, as a Potential Anti-Obesity Drug. J. Clin. Med..

[55] Trojan S.J., Hergenreder J. (2026). Mechanisms of Action of Beta-Agonists. Vet. Clin. Food Anim. Pract..

[56] Cui Y., Auclair H., He R., Zhang Q. (2024). GPCR-Mediated Regulation of Beige Adipocyte Formation: Implications for Obesity and Metabolic Health. Gene.

[57] Tran K.-V., Brown E.L., DeSouza T., Jespersen N.Z., Nandrup-Bus C., Yang Q., Yang Z., Desai A., Min S.Y., Rojas-Rodriguez R. (2020). Human Thermogenic Adipocyte Regulation by the Long Noncoding RNA LINC00473. Nat. Metab..

[58] Sun W., Dong H., Balaz M., Slyper M., Drokhlyansky E., Colleluori G., Giordano A., Kovanicova Z., Stefanicka P., Balazova L. (2020). snRNA-Seq Reveals a Subpopulation of Adipocytes That Regulates Thermogenesis. Nature.

[59] Xu H., Fu J.-L., Miao Y.-F., Wang C.-J., Han Q.-F., Li S., Huang S.-Z., Du S.-N., Qiu Y.-X., Yang J.-C. (2016). Prostaglandin E2 Receptor EP3 Regulates Both Adipogenesis and Lipolysis in Mouse White Adipose Tissue. J. Mol. Cell Biol..

[60] Tao X., Du R., Guo S., Feng X., Yu T., OuYang Q., Chen Q., Fan X., Wang X., Guo C. (2022). PGE2 -EP3 Axis Promotes Brown Adipose Tissue Formation through Stabilization of WTAP RNA Methyltransferase. EMBO J..

[61] Madsen L., Pedersen L.M., Lillefosse H.H., Fjaere E., Bronstad I., Hao Q., Petersen R.K., Hallenborg P., Ma T., De Matteis R. (2010). UCP1 Induction during Recruitment of Brown Adipocytes in White Adipose Tissue Is Dependent on Cyclooxygenase Activity. PLoS ONE.

[62] Tang E.H.C., Cai Y., Wong C.K., Rocha V.Z., Sukhova G.K., Shimizu K., Xuan G., Vanhoutte P.M., Libby P., Xu A. (2015). Activation of Prostaglandin E2-EP4 Signaling Reduces Chemokine Production in Adipose Tissue. J. Lipid Res..

[63] Bayindir I., Babaeikelishomi R., Kocanova S., Sousa I.S., Lerch S., Hardt O., Wild S., Bosio A., Bystricky K., Herzig S. (2015). Transcriptional Pathways in cPGI2-Induced Adipocyte Progenitor Activation for Browning. Front. Endocrinol..

[64] Ghandour R.A., Giroud M., Vegiopoulos A., Herzig S., Ailhaud G., Amri E.-Z., Pisani D.F. (2016). IP-Receptor and PPARs Trigger the Conversion of Human White to Brite Adipocyte Induced by Carbaprostacyclin. Biochim. Biophys. Acta (BBA)-Mol. Cell Biol. Lipids.

[65] Pisani D.F., Ghandour R.A., Beranger G.E., Le Faouder P., Chambard J.-C., Giroud M., Vegiopoulos A., Djedaini M., Bertrand-Michel J., Tauc M. (2014). The Ω6-Fatty Acid, Arachidonic Acid, Regulates the Conversion of White to Brite Adipocyte through a Prostaglandin/Calcium Mediated Pathway. Mol. Metab..

[66] Ghandour R.A., Colson C., Giroud M., Maurer S., Rekima S., Ailhaud G., Klingenspor M., Amri E.-Z., Pisani D.F. (2018). Impact of Dietary Ω3 Polyunsaturated Fatty Acid Supplementation on Brown and Brite Adipocyte Function. J. Lipid Res..

[67] Communi D., Horckmans M., Boeynaems J.-M. (2021). P2Y4, P2Y6 and P2Y11 Receptors: From the Early Days of Cloning to Their Function. Biochem. Pharmacol..

[68] Benoist L., Chadet S., Genet T., Lefort C., Heraud A., Danila M.D., Muntean D.M., Baron C., Angoulvant D., Babuty D. (2019). Stimulation of P2Y11 Receptor Protects Human Cardiomyocytes against Hypoxia/Reoxygenation Injury and Involves PKCε Signaling Pathway. Sci. Rep..

[69] Mathias L.S., Herman-de-Sousa C., Cury S.S., Nogueira C.R., Correia-de-Sá P., de Oliveira M. (2023). RNA-Seq Reveals That Anti-Obesity Irisin and Triiodothyronine (T3) Hormones Differentially Affect the Purinergic Signaling Transcriptomics in Differentiated Human Adipocytes. Biochim. Biophys. Acta (BBA)-Mol. Cell Biol. Lipids.

[70] Townsend L.K., Medak K.D., Knuth C.M., Peppler W.T., Charron M.J., Wright D.C. (2019). Loss of Glucagon Signaling Alters White Adipose Tissue Browning. FASEB J..

[71] Laker R.C., Egolf S., Will S., Lantier L., McGuinness O.P., Brown C., Bhagroo N., Oldham S., Kuszpit K., Alfaro A. (2025). GLP-1R/GCGR Dual Agonism Dissipates Hepatic Steatosis to Restore Insulin Sensitivity and Rescue Pancreatic β-Cell Function in Obese Male Mice. Nat. Commun..

[72] Regmi A., Aihara E., Christe M.E., Varga G., Beyer T.P., Ruan X., Beebe E., O’Farrell L.S., Bellinger M.A., Austin A.K. (2024). Tirzepatide Modulates the Regulation of Adipocyte Nutrient Metabolism through Long-Acting Activation of the GIP Receptor. Cell Metab..

[73] Lyons S.A., Lea M.B.S., Parikh M., Guo Z., Kagdi S., Bisnauth A.R., Pitino J.R., Ziai S., Mir N., Tyrrell A.D. (2025). Acute Exogenous Acyl-GIP Treatment Enhances Lipid Handling and Fatty Acid Oxidation by Involving Brown Fat. EMBO Rep..

[74] Jurado-Fasoli L., Martinez-Tellez B., Kohler I., Ruiz J.R., Osuna-Prieto F.J. (2026). Changes in Circulating Bile Acid Levels during Cold Exposure Are Associated with Brown Adipose Tissue in Humans: A Secondary Analysis from the ACTIBATE Study. J. Physiol. Biochem..

[75] Chan P.-C., Hung L.-M., Huang J.-P., Day Y.-J., Yu C.-L., Kuo F.-C., Lu C.-H., Tian Y.-F., Hsieh P.-S. (2022). Augmented CCL5/CCR5 Signaling in Brown Adipose Tissue Inhibits Adaptive Thermogenesis and Worsens Insulin Resistance in Obesity. Clin. Sci..

[76] Kurita K., Ishikawa K., Takeda K., Fujimoto M., Ono H., Kumagai J., Inoue H., Yokoh H., Yokote K. (2019). CXCL12-CXCR4 Pathway Activates Brown Adipocytes and Induces Insulin Resistance in CXCR4-Deficient Mice under High-Fat Diet. Sci. Rep..

[77] Yao L., Heuser-Baker J., Herlea-Pana O., Zhang N., Szweda L.I., Griffin T.M., Barlic-Dicen J. (2014). Deficiency in Adipocyte Chemokine Receptor CXCR4 Exacerbates Obesity and Compromises Thermoregulatory Responses of Brown Adipose Tissue in a Mouse Model of Diet-Induced Obesity. FASEB J..

[78] Takeda Y., Yoshikawa T., Dai P. (2024). Angiotensin II Participates in Mitochondrial Thermogenic Functions via the Activation of Glycolysis in Chemically Induced Human Brown Adipocytes. Sci. Rep..

[79] Vargas-Castillo A., Tobon-Cornejo S., Del Valle-Mondragon L., Torre-Villalvazo I., Schcolnik-Cabrera A., Guevara-Cruz M., Pichardo-Ontiveros E., Fuentes-Romero R., Bader M., Alenina N. (2020). Angiotensin-(1-7) Induces Beige Fat Thermogenesis through the Mas Receptor. Metabolism.

[80] Quesada-López T., Cereijo R., Turatsinze J.-V., Planavila A., Cairó M., Gavaldà-Navarro A., Peyrou M., Moure R., Iglesias R., Giralt M. (2016). The Lipid Sensor GPR120 Promotes Brown Fat Activation and FGF21 Release from Adipocytes. Nat. Commun..

[81] Lu D., He A., Tan M., Mrad M., El Daibani A., Hu D., Liu X., Kleiboeker B., Che T., Hsu F.-F. (2024). Liver ACOX1 Regulates Levels of Circulating Lipids That Promote Metabolic Health through Adipose Remodeling. Nat. Commun..

[82] Godlewski G., Jourdan T., Szanda G., Tam J., Cinar R., Harvey-White J., Liu J., Mukhopadhyay B., Pacher P., Ming Mo F. (2015). Mice Lacking GPR3 Receptors Display Late-Onset Obese Phenotype Due to Impaired Thermogenic Function in Brown Adipose Tissue. Sci. Rep..

[83] Sveidahl Johansen O., Ma T., Hansen J.B., Markussen L.K., Schreiber R., Reverte-Salisa L., Dong H., Christensen D.P., Sun W., Gnad T. (2021). Lipolysis Drives Expression of the Constitutively Active Receptor GPR3 to Induce Adipose Thermogenesis. Cell.

[84] Xiong Y., Xu Z., Li X., Wang Y., Zhao J., Wang N., Duan Y., Xia R., Han Z., Qian Y. (2024). Identification of Oleic Acid as an Endogenous Ligand of GPR3. Cell Res..

[85] Montgomery M.K., Osborne B., Brandon A.E., O’Reilly L., Fiveash C.E., Brown S.H.J., Wilkins B.P., Samsudeen A., Yu J., Devanapalli B. (2019). Regulation of Mitochondrial Metabolism in Murine Skeletal Muscle by the Medium-Chain Fatty Acid Receptor Gpr84. FASEB J..

[86] Sun X.-N., An Y.A., Paschoal V.A., de Souza C.O., Wang M.-Y., Vishvanath L., Bueno L.M., Cobb A.S., Nieto Carrion J.A., Ibe M.E. (2023). GPR84-Mediated Signal Transduction Affects Metabolic Function by Promoting Brown Adipocyte Activity. J. Clin. Investig..

[87] Kitada Y., Kajita K., Taguchi K., Mori I., Yamauchi M., Ikeda T., Kawashima M., Asano M., Kajita T., Ishizuka T. (2016). Blockade of Sphingosine 1-Phosphate Receptor 2 Signaling Attenuates High-Fat Diet-Induced Adipocyte Hypertrophy and Systemic Glucose Intolerance in Mice. Endocrinology.

[88] Gohlke S., Zagoriy V., Cuadros Inostroza A., Méret M., Mancini C., Japtok L., Schumacher F., Kuhlow D., Graja A., Stephanowitz H. (2019). Identification of Functional Lipid Metabolism Biomarkers of Brown Adipose Tissue Aging. Mol. Metab..

[89] Chakrabarty S., Bui Q., Badeanlou L., Hester K., Chun J., Ruf W., Ciaraldi T.P., Samad F. (2022). S1P/S1PR3 Signalling Axis Protects against Obesity-Induced Metabolic Dysfunction. Adipocyte.

[90] Ricciotti E., FitzGerald G.A. (2011). Prostaglandins and Inflammation. Arterioscler. Thromb. Vasc. Biol..

[91] García-Alonso V., Clària J. (2014). Prostaglandin E2 Signals White-to-Brown Adipogenic Differentiation. Adipocyte.

[92] Vegiopoulos A., Müller-Decker K., Strzoda D., Schmitt I., Chichelnitskiy E., Ostertag A., Berriel Diaz M., Rozman J., Hrabe de Angelis M., Nüsing R.M. (2010). Cyclooxygenase-2 Controls Energy Homeostasis in Mice by de Novo Recruitment of Brown Adipocytes. Science.

[93] Colson C., Batrow P.-L., Dieckmann S., Contu L., Roux C.H., Balas L., Vigor C., Fourmaux B., Gautier N., Rochet N. (2023). Effects of Fatty Acid Metabolites on Adipocytes Britening: Role of Thromboxane A2. Cells.

[94] Razzoli M., McGonigle S., Sahu B.S., Rodriguez P., Svedberg D., Rao L., Ruocco C., Nisoli E., Vezzani B., Frontini A. (2024). A Key Role for P2RX5 in Brown Adipocyte Differentiation and Energy Homeostasis. Adipocyte.

[95] Jaeckstein M.Y., Miegel L., Behrens J., Stähler T., Diercks B.-P., Heine M., Koch-Nolte F., Heeren J. (2025). The Purinergic Receptor P2X5 Modulates Glucose Metabolism and Expression of Thermogenic Genes in Brown Adipose Tissue. Int. J. Mol. Sci..

[96] Lee S.C., Vielhauer N.S., Leaver E.V., Pappone P.A. (2005). Differential Regulation of Ca^2+^ Signaling and Membrane Trafficking by Multiple P2 Receptors in Brown Adipocytes. J. Membr. Biol..

[97] Gruenbacher G., Gander H., Dobler G., Rahm A., Klaver D., Thurnher M. (2021). The Human G Protein-Coupled ATP Receptor P2Y11 Is a Target for Anti-Inflammatory Strategies. Br. J. Pharmacol..

[98] Conceição-Furber E., Coskun T., Sloop K.W., Samms R.J. (2022). Is Glucagon Receptor Activation the Thermogenic Solution for Treating Obesity?. Front. Endocrinol..

[99] Vaittinen M., Ilha M., Herbers E., Wagner A., Virtanen K.A., Pietiläinen K.H., Pirinen E., Pihlajamäki J. (2023). Liraglutide Demonstrates a Therapeutic Effect on Mitochondrial Dysfunction in Human SGBS Adipocytes in Vitro. Diabetes Res. Clin. Pract..

[100] Schmid A., Karrasch T., Schäffler A. (2023). The Emerging Role of Bile Acids in White Adipose Tissue. Trends Endocrinol. Metab..

[101] Broeders E.P.M., Nascimento E.B.M., Havekes B., Brans B., Roumans K.H.M., Tailleux A., Schaart G., Kouach M., Charton J., Deprez B. (2015). The Bile Acid Chenodeoxycholic Acid Increases Human Brown Adipose Tissue Activity. Cell Metab..

[102] Zietak M., Kozak L.P. (2016). Bile Acids Induce Uncoupling Protein 1-Dependent Thermogenesis and Stimulate Energy Expenditure at Thermoneutrality in Mice. Am. J. Physiol. Endocrinol. Metab..

[103] Li J., Liu Q., Xiong Y., Xu Y., Zhang J., Xia Y., Jing X., Zhang Z., Pang J., Huang C. (2026). Adipose TGR5 Deletion Promotes Hepatic Steatosis Through Decreasing Adiponectin Secretion in Mice. Diabetes.

[104] Chan P.-C., Hsieh P.-S. (2021). The Chemokine Systems at the Crossroads of Inflammation and Energy Metabolism in the Development of Obesity. Int. J. Mol. Sci..

[105] Proença A.B., Medeiros G.R., Reis G.D.S., Losito L.D.F., Ferraz L.M., Bargut T.C.L., Soares N.P., Alexandre-Santos B., Campagnole-Santos M.J., Magliano D.C. (2024). Adipose Tissue Plasticity Mediated by the Counterregulatory Axis of the Renin-Angiotensin System: Role of Mas and MrgD Receptors. J. Cell. Physiol..

[106] Evangelista F.S., Bartness T.J. (2023). Central Angiotensin 1-7 Triggers Brown Fat Thermogenesis. Physiol. Rep..

[107] Christian M. (2020). Elucidation of the Roles of Brown and Brite Fat Genes: GPR120 Is a Modulator of Brown Adipose Tissue Function. Exp. Physiol..

[108] Schilperoort M., van Dam A.D., Hoeke G., Shabalina I.G., Okolo A., Hanyaloglu A.C., Dib L.H., Mol I.M., Caengprasath N., Chan Y.-W. (2018). The GPR120 Agonist TUG-891 Promotes Metabolic Health by Stimulating Mitochondrial Respiration in Brown Fat. EMBO Mol. Med..

[109] Im H., Park J.-H., Im S., Han J., Kim K., Lee Y.-H. (2021). Regulatory Roles of G-Protein Coupled Receptors in Adipose Tissue Metabolism and Their Therapeutic Potential. Arch. Pharmacal Res..

[110] Valentine J.M., Ahmadian M., Keinan O., Abu-Odeh M., Zhao P., Zhou X., Keller M.P., Gao H., Yu R.T., Liddle C. (2022). B3-Adrenergic Receptor Downregulation Leads to Adipocyte Catecholamine Resistance in Obesity. J. Clin. Investig..

[111] Zhang N., Li Y. (2023). Receptor Tyrosine Kinases: Biological Functions and Anticancer Targeted Therapy. MedComm.

[112] Chen M., Zhu J., Luo H., Mu W., Guo L. (2024). The Journey towards Physiology and Pathology: Tracing the Path of Neuregulin 4. Genes Dis..

[113] Javankiani S., Bolandi S., Soleimani A., Meigoli M.S.S., Parsafar M., Safaei S., Esmailpour M., Nadimi S., Avval N.A., Fazayel S.M.A. (2025). MAPK Signaling Mediates Tamoxifen Resistance in Estrogen Receptor-Positive Breast Cancer. Mol. Cell. Biochem..

[114] Sakaguchi M. (2024). The Role of Insulin Signaling with FOXO and FOXK Transcription Factors. Endocr. J..

[115] Benvie A.M., Lee D., Jiang Y., Berry D.C. (2024). Platelet-Derived Growth Factor Receptor Beta Is Required for Embryonic Specification and Confinement of the Adult White Adipose Lineage. iScience.

[116] Boucher J., Mori M.A., Lee K.Y., Smyth G., Liew C.W., Macotela Y., Rourk M., Bluher M., Russell S.J., Kahn C.R. (2012). Impaired Thermogenesis and Adipose Tissue Development in Mice with Fat-Specific Disruption of Insulin and IGF-1 Signalling. Nat. Commun..

[117] Cao S., Pan Y., Tang J., Terker A.S., Arroyo Ornelas J.P., Jin G.-N., Wang Y., Niu A., Fan X., Wang S. (2022). EGFR-Mediated Activation of Adipose Tissue Macrophages Promotes Obesity and Insulin Resistance. Nat. Commun..

[118] Zeng F., Wang Y., Kloepfer L.A., Wang S., Harris R.C. (2018). ErbB4 Deletion Predisposes to Development of Metabolic Syndrome in Mice. Am. J. Physiol. Endocrinol. Metab..

[119] Chen Z., Zhang P., Liu T., Qiu X., Li S., Lin J.D. (2024). Neuregulin 4 Mediates the Metabolic Benefits of Mild Cold Exposure by Promoting Beige Fat Thermogenesis. JCI Insight.

[120] Tu W.-Z., Fu Y.-B., Xie X. (2019). RepSox, a Small Molecule Inhibitor of the TGFβ Receptor, Induces Brown Adipogenesis and Browning of White Adipocytes. Acta Pharmacol. Sin..

[121] Bahn Y.J., Wang Y., Dagur P., Scott N., Cero C., Long K.T., Nguyen N., Cypess A.M., Rane S.G. (2024). TGF-β Antagonism Synergizes with PPARγ Agonism to Reduce Fibrosis and Enhance Beige Adipogenesis. Mol. Metab..

[122] Constant B., Kamzolas I., Yang X., Guo J., Rodriguez-Fdez S., Mali I., Rodriguez-Cuenca S., Petsalaki E., Vidal-Puig A., Li W. (2025). Distinct Signalling Dynamics of BMP4 and BMP9 in Brown versus White Adipocytes. Sci. Rep..

[123] Long K.T., Cero C., Ali S.L., Nguyen N., Guarnieri A.R., Kim J.H., Bahn Y.J., Heymann J., Dreyfuss J.M., Rane S.G. (2026). Bone Morphogenetic Proteins 4 and 7 Increase Human White and Brown Adipocyte Thermogenic Capacity. JCI Insight.

[124] Iwayama T., Steele C., Yao L., Dozmorov M.G., Karamichos D., Wren J.D., Olson L.E. (2015). PDGFRα Signaling Drives Adipose Tissue Fibrosis by Targeting Progenitor Cell Plasticity. Genes Dev..

[125] Seki T., Hosaka K., Lim S., Fischer C., Honek J., Yang Y., Andersson P., Nakamura M., Näslund E., Ylä-Herttuala S. (2016). Endothelial PDGF-CC Regulates Angiogenesis-Dependent Thermogenesis in Beige Fat. Nat. Commun..

[126] Shamsi F., Piper M., Ho L.-L., Huang T.L., Gupta A., Streets A., Lynes M.D., Tseng Y.-H. (2021). Vascular Smooth Muscle-Derived Trpv1+ Progenitors Are a Source of Cold-Induced Thermogenic Adipocytes. Nat. Metab..

[127] Shi Z., Xiong S., Hu R., Wang Z., Park J., Qian Y., Wang J., Bhalla P., Velupally N., Song Q. (2024). The Notch-PDGFRβ Axis Suppresses Brown Adipocyte Progenitor Differentiation in Early Post-Natal Mice. Dev. Cell.

[128] Sun K., Kusminski C.M., Luby-Phelps K., Spurgin S.B., An Y.A., Wang Q.A., Holland W.L., Scherer P.E. (2014). Brown Adipose Tissue Derived VEGF-A Modulates Cold Tolerance and Energy Expenditure. Mol. Metab..

[129] Wang L., Jin J., Zhang N., Dai Y., Bai X., Li J., Yu Y., Shi X., Bai H., Yang Q. (2025). VEGFB Promotes Adipose Tissue Thermogenesis by Inhibiting Norepinephrine Clearance in Macrophages. Biochim. Biophys. Acta (BBA)-Mol. Basis Dis..

[130] Efthymiou V., Ding L., Balaz M., Sun W., Balazova L., Straub L.G., Dong H., Simon E., Ghosh A., Perdikari A. (2023). Inhibition of AXL Receptor Tyrosine Kinase Enhances Brown Adipose Tissue Functionality in Mice. Nat. Commun..

[131] Saltiel A.R., Kahn C.R. (2001). Insulin Signalling and the Regulation of Glucose and Lipid Metabolism. Nature.

[132] Townsend K.L., Tseng Y.-H. (2014). Brown Fat Fuel Utilization and Thermogenesis. Trends Endocrinol. Metab..

[133] Laplante M., Sabatini D.M. (2012). mTOR Signaling in Growth Control and Disease. Cell.

[134] Dai H.-B., Wang H.-Y., Wang F.-Z., Qian P., Gao Q., Zhou H., Zhou Y.-B. (2022). Adrenomedullin Ameliorates Palmitic Acid-Induced Insulin Resistance through PI3K/Akt Pathway in Adipocytes. Acta Diabetol..

[135] Man X.-F., Hu N., Tan S.-W., Tang H.-N., Guo Y., Tang C.-Y., Liu Y.-Q., Tang J., Zhou C.-L., Wang F. (2020). Insulin Receptor Substrate-1 Inhibits High-Fat Diet-Induced Obesity by Browning of White Adipose Tissue through miR-503. FASEB J..

[136] Gross D.N., van den Heuvel A.P.J., Birnbaum M.J. (2008). The Role of FoxO in the Regulation of Metabolism. Oncogene.

[137] Hotamisligil G.S. (2017). Inflammation, Metaflammation and Immunometabolic Disorders. Nature.

[138] BonDurant L.D., Potthoff M.J. (2018). Fibroblast Growth Factor 21: A Versatile Regulator of Metabolic Homeostasis. Annu. Rev. Nutr..

[139] Kharitonenkov A., Larsen P. (2011). FGF21 Reloaded: Challenges of a Rapidly Growing Field. Trends Endocrinol. Metab..

[140] Fisher F.M., Kleiner S., Douris N., Fox E.C., Mepani R.J., Verdeguer F., Wu J., Kharitonenkov A., Flier J.S., Maratos-Flier E. (2012). FGF21 Regulates PGC-1α and Browning of White Adipose Tissues in Adaptive Thermogenesis. Genes Dev..

[141] Abu-Odeh M., Zhang Y., Reilly S.M., Ebadat N., Keinan O., Valentine J.M., Hafezi-Bakhtiari M., Ashayer H., Mamoun L., Zhou X. (2021). FGF21 Promotes Thermogenic Gene Expression as an Autocrine Factor in Adipocytes. Cell Rep..

[142] Tanoue T., Nagayama M., Roochana A.J.A., Zimmerman S., Ashenberg O., Jain T., Igarashi R., Sasajima S., Takeshita K., Hetherington N. (2026). Microbiota-Mediated Induction of Beige Adipocytes in Response to Dietary Cues. Nature.

[143] Yarden Y., Pines G. (2012). The ERBB Network: At Last, Cancer Therapy Meets Systems Biology. Nat. Rev. Cancer.

[144] Liu Y., Chen M. (2022). Neuregulin 4 as a Novel Adipokine in Energy Metabolism. Front. Physiol..

[145] Comas F., Martínez C., Sabater M., Ortega F., Latorre J., Díaz-Sáez F., Aragonés J., Camps M., Gumà A., Ricart W. (2019). Neuregulin 4 Is a Novel Marker of Beige Adipocyte Precursor Cells in Human Adipose Tissue. Front. Physiol..

[146] Liao J., Wu T., Zhang Q., Shen P., Huang Z., Wang J., Zhang P., Lin S., Pi J., Zhang N. (2026). TGF-β/BMP Signaling in Skeletal Biology: Molecular Mechanisms, Regulatory Networks, and Therapeutic Implications in Development, Regeneration, and Disease. Bone Res..

[147] Tavares A.H., Connelly S.P., Maksat D., Zheng J., Rabhi N., Layne M.D. (2025). TGF-B1-Dependent Expression of FOXS1 Attenuates Adipogenic Potential and Enhances a Myofibroblast Cellular Phenotype. J. Biol. Chem..

[148] Qian S., Tang Y., Tang Q.-Q. (2021). Adipose Tissue Plasticity and the Pleiotropic Roles of BMP Signaling. J. Biol. Chem..

[149] Gao Z., Daquinag A.C., Su F., Snyder B., Kolonin M.G. (2018). PDGFRα/PDGFRβ Signaling Balance Modulates Progenitor Cell Differentiation into White and Beige Adipocytes. Development.

[150] Sung H.-K., Doh K.-O., Son J.E., Park J.G., Bae Y., Choi S., Nelson S.M.L., Cowling R., Nagy K., Michael I.P. (2013). Adipose Vascular Endothelial Growth Factor Regulates Metabolic Homeostasis through Angiogenesis. Cell Metab..

[151] Qian C., Wang C., Liu F. (2025). AXL in Cardiovascular Diseases: Pathophysiological Insights and Clinical Perspectives. Pathol. Res. Pract..

[152] Kajimura S., Spiegelman B.M., Seale P. (2015). Brown and Beige Fat: Physiological Roles beyond Heat Generation. Cell Metab..

[153] Ahmadian M., Suh J.M., Hah N., Liddle C., Atkins A.R., Downes M., Evans R.M. (2013). PPARγ Signaling and Metabolism: The Good, the Bad and the Future. Nat. Med..

[154] Kim H.-Y., Jang H.-J., Muthamil S., Shin U.C., Lyu J.-H., Kim S.-W., Go Y., Park S.-H., Lee H.G., Park J.H. (2024). Novel Insights into Regulators and Functional Modulators of Adipogenesis. Biomed. Pharmacother..

[155] Rachid T.L., Silva-Veiga F.M., Graus-Nunes F., Bringhenti I., Mandarim-de-Lacerda C.A., Souza-Mello V. (2018). Differential Actions of PPAR-α and PPAR-β/δ on Beige Adipocyte Formation: A Study in the Subcutaneous White Adipose Tissue of Obese Male Mice. PLoS ONE.

[156] Hondares E., Rosell M., Díaz-Delfín J., Olmos Y., Monsalve M., Iglesias R., Villarroya F., Giralt M. (2011). Peroxisome Proliferator-Activated Receptor α (PPARα) Induces PPARγ Coactivator 1α (PGC-1α) Gene Expression and Contributes to Thermogenic Activation of Brown Fat: Involvement of PRDM16. J. Biol. Chem..

[157] Jiang T., Su D., Ke J., Dai X., Wang M., Wang Y., Zhan S., Zhong T., Guo J., Li L. (2025). A Distal Enhancer of Pparα Regulates Thermogenesis and Mitochondrial Function in Brown Fat. PLoS Genet..

[158] Shin K.C., Huh J.Y., Ji Y., Han J.S., Han S.M., Park J., Nahmgoong H., Lee W.T., Jeon Y.G., Kim B. (2022). VLDL-VLDLR Axis Facilitates Brown Fat Thermogenesis through Replenishment of Lipid Fuels and PPARβ/δ Activation. Cell Rep..

[159] Korach-André M., Archer A., Barros R.P., Parini P., Gustafsson J.-Å. (2011). Both Liver-X Receptor (LXR) Isoforms Control Energy Expenditure by Regulating Brown Adipose Tissue Activity. Proc. Natl. Acad. Sci. USA.

[160] Miao Y., Warner M., Gustafsson J.-K. (2016). Liver X Receptor β: New Player in the Regulatory Network of Thyroid Hormone and “browning” of White Fat. Adipocyte.

[161] Kim S.H., Park W.Y., Kim B., Kim J.-H., Song G., Park J.Y., Jiao W., Jung S.J., Ahn K.S., Kwak H.J. (2025). FXR-ApoC2 Pathway Activates UCP1-Mediated Thermogenesis by Promoting the Browning of White Adipose Tissues. J. Biol. Chem..

[162] Liu S., Shen S., Yan Y., Sun C., Lu Z., Feng H., Ma Y., Tang Z., Yu J., Wu Y. (2022). Triiodothyronine (T3) Promotes Brown Fat Hyperplasia via Thyroid Hormone Receptor α Mediated Adipocyte Progenitor Cell Proliferation. Nat. Commun..

[163] Roth L., Hoffmann A., Hagemann T., Wagner L., Strehlau C., Sheikh B., Donndorf L., Ghosh A., Noé F., Wolfrum C. (2024). Thyroid Hormones Are Required for Thermogenesis of Beige Adipocytes Induced by Zfp423 Inactivation. Cell Rep..

[164] Murholm M., Isidor M.S., Basse A.L., Winther S., Sørensen C., Skovgaard-Petersen J., Nielsen M.M., Hansen A.S., Quistorff B., Hansen J.B. (2013). Retinoic Acid Has Different Effects on UCP1 Expression in Mouse and Human Adipocytes. BMC Cell Biol..

[165] Lee N.H., Choi M.J., Yu H., Kim J.I., Cheon H.G. (2022). Adapalene Induces Adipose Browning through the RARβ-P38 MAPK-ATF2 Pathway. Arch. Pharm. Res..

[166] Chen D., Woraikat S., Guo X., Yang F., Tang C., He F., Qian K. (2025). Retinoid X Receptor γ Interacts with Peroxisome Proliferator-Activated Receptor-γ to Promote Browning during Adipose Tissue Differentiation. Adipocyte.

[167] Nie B., Nie T., Hui X., Gu P., Mao L., Li K., Yuan R., Zheng J., Wang H., Li K. (2017). Brown Adipogenic Reprogramming Induced by a Small Molecule. Cell Rep..

[168] Sveidahl Johansen O., McIntyre R.L., Rahbani J.F., Zhang Q., Scholtes C., Lagarde D.M., Billon C., Côté I., Delgado-Martin M., Tandio D. (2026). Cold Exposure Induces the Constitutively Active Thermogenic Receptor, GPR3, via ERRα and ERRγ. Mol. Metab..

[169] Choe H.J., Lee J.S., Park J.Y., Lee S.-A., Park Y.J., Chung S.S., Park K.S. (2025). SENP2 Regulates UCP1-Dependent Thermogenesis in Brown Adipocytes via deSUMOylation of ERRα. Exp. Mol. Med..

[170] Zhao S., Nie T., Li L., Long Q., Gu P., Zhang Y., Sun W., Lin Z., Liu Q., Qi Y. (2023). Androgen Receptor Is a Negative Regulator of PRDM16 in Beige Adipocyte. Adv. Sci..

[171] Harada N., Kubo K., Onishi T., Kitakaze T., Goto T., Inui H., Yamaji R. (2022). Androgen Receptor Suppresses β-Adrenoceptor-Mediated CREB Activation and Thermogenesis in Brown Adipose Tissue of Male Mice. J. Biol. Chem..

[172] Martín F.M., Alzamendi A., Harnichar A.E., Castrogiovanni D., Zubiría M.G., Spinedi E., Giovambattista A. (2023). Role of Glucocorticoid Receptor (GR) in White Adipose Tissue Beiging. Life Sci..

[173] Kroon J., Koorneef L.L., van den Heuvel J.K., Verzijl C.R.C., van de Velde N.M., Mol I.M., Sips H.C.M., Hunt H., Rensen P.C.N., Meijer O.C. (2018). Selective Glucocorticoid Receptor Antagonist CORT125281 Activates Brown Adipose Tissue and Alters Lipid Distribution in Male Mice. Endocrinology.

[174] Urbanet R., Nguyen Dinh Cat A., Feraco A., Venteclef N., El Mogrhabi S., Sierra-Ramos C., Alvarez de la Rosa D., Adler G.K., Quilliot D., Rossignol P. (2015). Adipocyte Mineralocorticoid Receptor Activation Leads to Metabolic Syndrome and Induction of Prostaglandin D2 Synthase. Hypertension.

[175] Chen C.-M., Meng X.-Q., Zhu H., Liu T., Liu Y., Zhou L.-J., Zhu G.-D., Chen X.-B., Guo X.-G., Duan S.-Z. (2023). Brown Adipocyte Mineralocorticoid Receptor Deficiency Impairs Metabolic Regulation in Diet-Induced Obese Mice. J. Lipid Res..

[176] Takeda Y., Dai P. (2020). A Developed Serum-Free Medium and an Optimized Chemical Cocktail for Direct Conversion of Human Dermal Fibroblasts into Brown Adipocytes. Sci. Rep..

[177] Takeda Y., Yoshikawa T., Dai P. (2021). Transcriptome Analysis Reveals Brown Adipogenic Reprogramming in Chemical Compound-Induced Brown Adipocytes Converted from Human Dermal Fibroblasts. Sci. Rep..

[178] Dixon E.D., Nardo A.D., Claudel T., Trauner M. (2021). The Role of Lipid Sensing Nuclear Receptors (PPARs and LXR) and Metabolic Lipases in Obesity, Diabetes and NAFLD. Genes.

[179] Liu J., Wang Y., Lin L. (2019). Small Molecules for Fat Combustion: Targeting Obesity. Acta Pharm. Sin. B.

[180] Mullur R., Liu Y.-Y., Brent G.A. (2014). Thyroid Hormone Regulation of Metabolism. Physiol. Rev..

[181] Sabatino L., Vassalle C. (2025). Thyroid Hormones and Metabolism Regulation: Which Role on Brown Adipose Tissue and Browning Process?. Biomolecules.

[182] Oelkrug R., Harder L., Pedaran M., Hoffmann A., Kolms B., Inderhees J., Gachkar S., Resch J., Johann K., Jöhren O. (2023). Maternal Thyroid Hormone Receptor β Activation in Mice Sparks Brown Fat Thermogenesis in the Offspring. Nat. Commun..

[183] Zekri Y., Guyot R., Suñer I.G., Canaple L., Stein A.G., Petit J.V., Aubert D., Richard S., Flamant F., Gauthier K. (2022). Brown Adipocytes Local Response to Thyroid Hormone Is Required for Adaptive Thermogenesis in Adult Male Mice. eLife.

[184] Zhao P., Fang H., Elgendy B., Hegazy L. (2026). Structural Pharmacology of Estrogen-Related Receptors. ACS Pharmacol. Transl. Sci..

[185] Kounatidis D., Vallianou N.G., Rebelos E., Kouveletsou M., Kontrafouri P., Eleftheriadou I., Diakoumopoulou E., Karampela I., Tentolouris N., Dalamaga M. (2025). The Many Facets of PPAR-γ Agonism in Obesity and Associated Comorbidities: Benefits, Risks, Challenges, and Future Directions. Curr. Obes. Rep..

